# Revisiting the hazards of hazard ratios through simulations and case studies

**DOI:** 10.1007/s10654-025-01245-6

**Published:** 2025-07-03

**Authors:** Michal Abrahamowicz, Marie-Eve Beauchamp, Emily K. Roberts, Jeremy M. G. Taylor

**Affiliations:** 1 Department of Epidemiology, Biostatistics and Occupational Health, McGill University, Montreal, QC Canada; 2 Centre for Outcomes Research and Evaluation (CORE), Research Institute of the McGill University Health Centre, Montreal, QC Canada; 3 Department of Biostatistics, University of Iowa, Iowa City, IA USA; 4 Department of Biostatistics, University of Michigan, Ann Arbor, MI USA

**Keywords:** Built-in selection bias, Cox model, Proportional hazards, Time-dependent effects, Depletion of susceptible, Frailty

## Abstract

The hazard has been a central concept in the analysis and interpretation of time-to-event data for over 50 years. At any follow-up time, the hazard is the probability of the event happening in the next unit of time amongst those still at risk. Hazard ratios (HRs) between groups are frequently used to quantify the exposure/treatment’s association with the failure time. In a highly cited paper, Hernán criticized HRs, asserting that their decreases over time may reflect simply a built-in selection bias, induced by an unmeasured susceptibility, and should not be interpreted as genuine changes in treatment effect. Hernán supports his arguments mainly by the results of a hormone therapy trial, where the HR for coronary heart events decreased largely during follow-up, with hazards crossing from harmful to protective treatment effect. However, he did not present simulations or algebraic derivations to demonstrate that these changes may reflect just an unmeasured susceptibility. We use simulations and real-world case studies to systematically explore this issue. The first simulation series reveals how the underestimation bias and changes over time in Cox proportional hazards model-based HRs depend on the joint impact of susceptibility on the hazard, its distribution, and the incidence of events; with important bias toward the null occurring only for a very strong susceptibility. Further simulations mimic the hormone therapy trial highlighted by Hernán, to demonstrate that the reported bias and crossing hazards are extremely unlikely to reflect just an unmeasured susceptibility, which suggests some biological reasons for decaying treatment HR, possibly including decreasing treatment adherence. Finally, we present real-world examples of interpretable and clinically plausible time-dependent HRs in cancer research. In conclusion, results of our simulations and real-world case studies suggest that concerns about HR limitations may be overstated, and we encourage researchers to model time-dependent HRs and consider potential biological and clinical causes thereof.

**Supplementary Information:**

The online version contains supplementary material available at 10.1007/s10654-025-01245-6.

## Introduction

Interpretation of the results of complex analyses in studies of human health requires combining (i) an understanding of the relevant analytical issues with (ii) context-dependent substantive insights, and may require considering alternative plausible explanations. In this article, we focus on highly debated issues of hazard ratios and illustrate how carefully designed statistical simulations and real-world examples may help with (i) and (ii).

In 2010, Hernán published a very influential commentary “Hazards of hazard ratios” [1], which focuses on the built-in selection bias inherent in the estimation of hazard ratios (HRs). The cornerstone of Hernán’s arguments is that the presence of an unobserved risk factor, a “susceptibility” or “frailty”, will automatically induce systematic biases in the estimated effect of a truly harmful exposure/treatment. These interrelated biases include: (a) an underestimation of the Cox model-based HR (constrained a priori to be constant over time by the underlying proportional hazards (PH) assumption), and (b) a gradual decrease toward the null of period-specific HRs with increasing follow-up duration. Specifically, Hernán argues that biases in (b) may eventually produce a spurious evidence of (c) the “crossing hazards” phenomenon, with the estimated exposure HR gradually falling from initial risk increase (HR > 1.0) to below 1.0 later in the follow-up [1]. Thus, even if the exposure is truly harmful throughout follow-up, the estimated HR may incorrectly suggest a long-term *protective* effect, i.e. *reduced* risk [1].

Hernán’s commentary has received > 800 citations in the past 14 years, many of which explicitly state that they did not rely on Cox PH model hazard ratio estimates because of the concerns about built-in selection bias [2–6]. Our work was partly motivated by our belief that some of these concerns may be disproportionally serious relative to the limited strength of the empirical evidence provided by Hernán [1], which is restricted to the results of a single randomized-controlled trial (RCT) of hormone therapy by Manson et al. [7]. We recognize that phenomena (a) and (b) are generally well established in the statistical survival analytical literature, since the mid-1980’s [8–10], indicating that Cox PH model, and its flexible extensions, do not yield unbiased estimates of the causal treatment/exposure effects. In contrast, the expected strength of the resulting bias toward the null, and the plausibility of, and the conditions necessary for the unmeasured susceptibility to induce “crossing hazards”, are less clear. For the RCT discussed by Hernán, the point estimates of year-specific HRs for combined hormone therapy vs. placebo gradually decreased from 1.81 in the 1 st year to about 0.70 after six years [7]. However, Hernán did not investigate whether this large decrease in the HR over time could be entirely explained by unobserved susceptibility, using either algebraic developments or simulations [1]. Furthermore, it is important to note that, in the original publication, the 95% confidence interval (CI) for HR = 0.70 in years 6–8 of the trial was (0.42 to 1.14), i.e. did include 1.0 [7].

Interestingly, a recent review of 27 large RCTs, a majority of which focused on cardiovascular events, *failed* to find evidence of built-in selection bias leading to a systematic attenuation of the treatment HRs [11]. Accordingly, the authors concluded that the existence of a strong unmeasured susceptibility for major cardiovascular outcomes, similar to those assessed by Manson et al. [7], is unlikely [11]. This finding reinforces the need to consider alternative explanations for Manson et al.’s RCT results.

Recognizing that, for different reasons, HRs may often vary over time, in the past three decades statisticians developed several flexible extensions of the Cox model that allow for both: (i) testing the PH hypothesis and (ii) modeling the time-dependent (TD) effects that describe how the HR changes during follow-up (e.g., [12–17]). Real-world applications of these flexible TD models yielded many examples of systematic, clinically or biologically plausible, changes over time in adjusted HRs for various risk/prognostic factors, exposures and treatments (e.g., [17–26]). For example, in a large study of survival of more than 40,000 dialysis patients on a waiting list for a kidney transplant, having received a cadaveric transplant was associated with much higher early mortality in the first weeks after the transplant (adjusted aHR = 2.8) but a large reduction of mortality after one year or later (aHR = 0.3) [27]. Importantly, the authors provide convincing arguments for the clinical plausibility of this strong time-varying effect, leading to “crossing hazards” [27]. Interestingly, Prentice and Aragaki [28] recently reanalyzed the Women’s Health Initiative RCT data discussed by Hernan [1] to illustrate how time-dependent HRs reflect a contrast between survival of the two treatment groups and may help understand its biological basis. Other examples involving cardiovascular events include the decreasing protective effect of aspirin, due to the gradual development of resistance to treatment [19], or crossing hazards for the short- vs. long-term impact of an anti-retroviral drug [29, 30]. Similarly, a major curative surgery may increase short-term mortality, due to post-surgical complications, but *decrease* it in the long-term [23, 31]. Finally, especially in intention-to-treat analyses of RCTs, the observed treatment effect may gradually decay toward the null simply because of decreasing treatment adherence (e.g., [32]). In all these situations, the estimated period-specific treatment HRs will generally decrease with longer follow-up, possibly with crossing hazards, but main plausible reasons involve mechanisms other than unmeasured susceptibility. Thus, it is important to systematically assess if and to what extent an unmeasured susceptibility may explain the results of the hormone therapy RCT discussed by Hernan [1].

Other studies have documented that Cox PH model estimates of treatment effects differ from the true value when a susceptibility (or frailty) variable is omitted (e.g., [33–38]). While the depletion over time of susceptible individuals, and its consequence on HR estimates, are also discussed and examined in a limited number of simulation studies (e.g., [33–38]), published simulation results are not specific enough to determine if this phenomenon could produce the pattern of time-varying changes in HRs reported by Manson et al. [7].

The current manuscript has three main objectives, the first two being addressed by simulations. First, we systematically explore how the bias induced in the estimated treatment effect by a failure to adjust for an unmeasured susceptibility, and the resulting spurious time-varying changes in year-specific treatment HRs, depend on the distribution of susceptibility and its impact on the hazard, as well as on the incidence rate of the outcome. Secondly, we present simulations that closely mimic the hormone therapy RCT discussed by Hernán [1], to assess to what extent, and under what assumptions, the empirical results of that trial could simply reflect the presence of an unmeasured susceptibility. The third objective is to illustrate the clinical plausibility, and interpretability, of flexible estimates of time-dependent effects of important prognostic factors or treatments in real-world analyses. These examples emphasize the advantages of exploring and reporting systematic changes in the HR during follow-up that likely reflect the underlying clinical or biological processes rather than merely a built-in selection bias due to a depletion of susceptibles.

## General simulation methods

This section describes the design and methods of simulations reported in Sects."Simulation results assessing the impact of unobserved susceptibility"and"Targeted simulations to mimic the RCT discussed by Hernán". We follow the ADEMP (Aims, Data generation, Estimands, Methods, Performance measures) structure [39] to describe our simulation studies.

*Aims:* All simulations mimicked a hypothetical randomized trial of the association between a time-invariant binary treatment *A* and the hazard of an adverse event. The overall aims were to assess the impact of unobserved susceptibility *S*, an unmeasured risk factor, on the Cox PH model-based estimates of the overall and time-dependent year-specific HRs for treatment; and explore how this impact varies depending on relevant assumptions and design parameters.

*Data Generation:* Across simulations, we generated sample sizes of either *N* = 4,000 or *N* = 16,000, the latter being similar to the hormone therapy RCT [7]. Individual binary treatment indicators were generated from the Bernoulli distribution with P(*A* = 1) = 0.5. For each participant, sex (P(female) = 0.5) and age (truncated normal distribution with mean 60 and range 20–100) were generated independently of each other and of treatment. Susceptibility *S* was generated, independently of treatment and covariates, from different distributions depending on the scenario (see specific subsections below for details).

Individual event times were generated from the following PH model, depending on treatment, susceptibility, age (HR = 1.04 for 1 year increase), and sex (HR = 0.8 for female vs. male):

1 $$\lambda \left(t|A,S, age, sex\right)={\lambda }_{0}\text{exp}\left[{\beta }_{A}A+{\beta }_{s}S+\text{log}(1.04)\left(age-60\right)+\text{log}(0.8)sex\right]$$

True log HRs ${\beta }_{A}$ for treatment, which can be considered an individual level causal effect [33, 40], and susceptibility ${\beta }_{s}$ varied across scenarios, as described later. Administrative censoring at the end of the trial was either (a) after 10 years (Sect."Simulation results assessing the impact of unobserved susceptibility"), or (b) after the observed total number of events reached 335 (Sect."Targeted simulations to mimic the RCT discussed by Hernán"), to match the incidence reported for the Manson et al.’s trial [7]. The constant baseline hazard (*λ*_0_ in Eq. (1)) was fixed to generate a pre-specified baseline survival probability in the untreated after 5 years, which (a) varied across scenarios of Sect."Simulation results assessing the impact of unobserved susceptibility", or (b) was fixed to 0.999 in Sect."Targeted simulations to mimic the RCT discussed by Hernán".

*Estimands and Methods:* Our main focus is on the estimated log(HR) for treatment *A* in the multivariable Cox PH model that adjusts for age and sex but *not* for susceptibility *S*. This estimand is a population-averaged quantity, expected to differ from ${\beta }_{A}$. For each simulated sample, we estimated both (i) the overall log(HR) from the Cox PH model fit to all data, and (ii) year-specific log(HR)’s, similar to those reported by Manson et al. [7] and Hernán [1]. The latter were obtained by fitting a separate multivariable Cox model for each year *t* during follow-up, including only participants who had no event until the beginning of year *t;* and right-censoring, at the year’s end, all those who had no event during year *t*. In addition, for selected scenarios we fit a flexible extension of the Cox PH model that used unpenalized quadratic regression splines to estimate a smooth, continuous function of follow-up time, describing the time-dependent log(HR)(*t*) for treatment [41, 42].

*Performance measures:* Results of each scenario were summarized across 1,000 independent simulated samples. For the Cox PH model fit to all data we report the following measures for the adjusted log(HR) for treatment: bias (difference between the mean of 1,000 estimates and the true value) and relative bias (bias divided by the true log(HR)). Furthermore, we (i) report relative biases of year-specific HR estimates, and (ii) for selected scenarios, compare the distributions of susceptibility *S* between treated (*A* = 1) *versus* untreated (*A* = 0) participants who remain at risk until the beginning of each year. To explore by how much individual sample estimates may underestimate the true HR for treatment in the presence of unobserved susceptibility *S*, for both the overall Cox PH model and selected year-specific models, in addition to the mean of the 1,000 HR estimates, we show the minimum and 5th percentile of their distribution for selected simulation scenarios of Sect."Targeted simulations to mimic the RCT discussed by Hernán". For flexible modeling of time-dependent treatment effects, we display graphically the estimates, and report the proportion of simulated samples for which the PH assumption was rejected by a model-based likelihood ratio test at α = 0.05 [41, 42].

Simulations were performed using R (version 4.4.1).

## Simulation results assessing the impact of unobserved susceptibility

### Objectives

The first series of simulations explored how Cox model estimates of both the overall log(HR) for treatment *A*, estimated under the PH assumption, and the year-specific time-dependent log(HR)’s were affected by the unobserved strong susceptibility *S,* under different assumptions. Specifically, we explored the impact of the (i) strength of the association between the unobserved susceptibility *S* and the outcome, (ii) strength of the treatment-outcome association, (iii) cumulative incidence of the outcome during follow-up, and (iv) distribution of *S*. In different scenarios, susceptibility *S* was generated as either a binary variable with different prevalences (Subsect."Results for binary susceptibility"), or a continuous normally distributed variable (Subsect."Results for continuous susceptibility"). In all simulations of this section the true treatment effect was constant over time, i.e. the generated data were fully consistent with the PH assumption.

### Results for binary susceptibility

Table 1 summarizes the results of simulations in which, using the general methods of Sect."General simulation methods", we generated a binary indicator of unobserved susceptibility *S*, e.g. the presence of an unmeasured genetic mutation, from Bernoulli distributions with different prevalences P(*S* = 1) for alternative scenarios (column 4).

**Table 1:** Results of simulations with a binary susceptibility *S*

Scenario	*N*	5-year baseline survival	P(*S* = 1)	True HR(*S*)	True HR(*A*)	Mean cumulative incidence, %	Overall relative bias, %	Year-specific (year 2) relative bias, %	Year-specific (year 4) relative bias, %	Year-specific (year 6) relative bias, %	Year-specific (year 8) relative bias, %	Year-specific (year 10) relative bias, %
Col. 1	Col. 2	Col. 3	Col. 4	Col. 5	Col. 6	Col. 7	Col. 8	Col. 9	Col 10	Col. 11	Col. 12	Col. 13
1	4,000	0.9	0.5	2	2.0	34.3	−2.4	−1.5	−1.2	−2.5	−4.3	−5.0
2	4,000	0.9	0.5	3	2.0	40.6	−7.2	−1.5	−5.7	−10.0	−12.8	−15.1
3	4,000	0.9	0.5	4	2.0	45.2	−12.4	−4.5	−10.6	−15.1	−23.0	−26.5
4	4,000	0.9	0.5	5	2.0	48.7	−17.8	−6.8	−16.2	−25.5	−30.9	−38.1
5	4,000	0.9	0.5	10	2.0	57.4	−38.2	−20.3	−47.0	−66.0	−74.7	−76.9
6	4,000	0.9	0.5	15	2.0	60.4	−49.1	−34.9	−75.6	−93.0	−94.9	−81.1
7	4,000	0.9	0.5	20	2.0	61.5	−54.8	−48.8	−99.7	−106.4	−89.6	−67.2
8	4,000	0.9	0.5	25	2.0	62.1	−58.3	−64.0	−115.0	−110.5	−78.5	−51.9
9	16,000	0.9	0.5	25	2.0	62.1	−58.5	−64.1	−115.6	−109.0	−78.9	−50.0
10	4,000	0.9	0.5	10	1.3	53.3	−34.3	−13.8	−38.0	−54.0	−65.3	−73.5
11	4,000	0.9	0.5	10	1.5	54.7	−36.2	−17.4	−39.8	−60.3	−73.1	−80.5
12	4,000	0.9	0.5	10	4.0	63.6	−40.3	−30.4	−59.7	−69.0	−65.9	−59.4
13	4,000	0.9	0.5	10	6.0	67.1	−40.3	−37.2	−62.2	−63.8	−56.6	−49.8
14	4,000	0.9	0.25	10	2.0	41.4	−31.3	−20.8	−39.4	−46.2	−44.9	−42.1
15	4,000	0.9	0.1	10	2.0	31.8	−17.1	−14.0	−22.0	−21.3	−20.6	−15.7
16	4,000	0.9	0.025	10	2.0	27.0	−5.0	−4.2	−6.1	−4.1	−4.5	−1.6
17	4,000	0.97	0.5	10	2.0	31.5	−17.1	−4.6	13.8	−21.5	−29.4	−38.5
18	4,000	0.97	0.25	10	2.0	19.9	−18.3	−6.2	−15.5	−21.0	−31.2	−32.9
19	4,000	0.97	0.1	10	2.0	13.0	−12.0	−4.3	−7.7	−14.2	−16.6	−17.9
20	4,000	0.97	0.025	10	2.0	9.5	−4.7	−0.6	−3.5	−3.7	−5.3	−2.1
21	4,000	0.7	0.5	10	2.0	79.6	−40.5	−59.8	−72.5	−45.9	−25.1	−14.7
22	4,000	0.7	0.25	10	2.0	69.6	−26.5	−43.5	−35.2	−18.3	−9.0	−2.7
23	4,000	0.7	0.1	10	2.0	63.9	−12.5	−22.5	−12.5	−6.8	−4.8	−2.4
24	4,000	0.7	0.025	10	2.0	60.6	−3.0	−4.6	−2.5	−0.4	−0.7	1.0
25	4,000	0.99	0.5	10	2.0	13.7	−6.0	−1.2	−2.5	−6.2	−5.7	−10.6
26	4,000	0.99	0.25	10	2.0	8.3	−5.8	1.1	−2.8	−3.7	−7.4	−11.6

*Col*. Column, *HR* hazard ratio

Bias in year-specific log(HR) estimates for exposure varies considerably across columns 9–13 of Table 1, indicating a violation of the PH assumption. Thus, the overall estimate from the Cox model fit to data for the entire follow-up, which imposes the PH assumption (column 8), is just the average of year-specific estimates. As discussed below, different aspects of simulated scenarios affect the strength of the bias in the overall (column 8) and year-specific (columns 9–13) estimates similarly, but the latter provide additional insights regarding changes over time in relative bias.

Scenarios 1–9 confirm that the underestimation bias increases steadily with stronger impact of susceptibility *S.* However, as long as HR(*S*) does not exceed 5, the relative bias is not large, < 20% and < 40% for the overall and year-specific estimates, respectively (scenarios 1–4). Large biases were obtained only under extreme assumptions of HR(*S*) ≥ 15, almost never reported for any exposures in real-world epidemiological studies. Even then, the overall log(HR) exposure estimates are reduced, on average, by less than 60% (scenarios 6–9), i.e. still indicate a risk increase. Interestingly, even the highest relative bias of 58.5% in Table 1 (scenario 9 with an extremely strong HR(*S*) = 25) is still lower than 63.7%, corresponding to the discrepancy, reported for the original RCT [7], between the overall log HR of 0.215 = log(1.24) versus the 1 st year estimate of 0.593 = log(1.81), considered unbiased by Hernán [1]. However, the corresponding year-specific estimates for years 4–8 approach or even exceed 100% bias (columns 10–12), demonstrating that an extremely strong susceptibility may induce a spurious evidence of crossing hazards, where a truly harmful exposure has estimated HR < 1 over some portion of follow-up. Interestingly, for all these extreme scenarios, the changes are *not* monotone and year-specific estimates increase, often substantially, in late years 8 and 10 (scenarios 7–9).

To explore the impact of other factors, in scenarios 10–26, we considered a very strong susceptibility (HR(*S*) = 10). Across scenarios 5 and 10–13, which differ only in terms of true exposure HR, the *relative* bias of the overall HR remains quite stable, around 34–40% (column 8). However, for year-specific estimates, with longer follow-up, bias increases monotonically for weaker exposure effects (scenarios 5, 10, and 11) but shows an up-and-down pattern for strong exposure HR (scenarios 12–13).

Generally, the bias decreases with lower prevalence of susceptibility (Table 1). For all scenarios with P(*S*) ≤ 0.25 or P(*S*) ≤ 0.1, the bias in year-specific estimates never exceeds 47% or 22%, respectively (scenarios 14–16, 18–20, 22–24, and 26). Thus, even a very strong (HR(*S*) = 10) but rare unmeasured risk factor (e.g., a rare genetic mutation) cannot induce very strong biases. Bias tends also to decrease for lower cumulative incidence (column 7 of Table 1). If less than 15% of the participants have events, bias toward the null in year-specific HRs never exceeds 18% (scenarios 19–20 and 25–26). Thus, in studies of rare events, with above 85% censoring, even a susceptibility associated which a tenfold hazard increase is very unlikely to induce important biases.

Based on selected scenarios of Table 1, Supplementary Fig. S1 illustrates how the relative bias of the overall log(HR) for treatment varies across different combinations of the prevalence of susceptibility P(*S* = 1) (column 4) and baseline survival rate (column 3), when *S* has a very strong impact on the hazard. It illustrates the complex joint effects of these factors, where the impact of the prevalence of *S* may depend on the survival rate and vice versa. Supplementary Material section S1 provides more detailed comments on Supplementary Fig. S1.

Section S2 of Supplementary Material and Supplementary Tables S1 and S2 present results for additional scenarios with a high prevalence of *S*.

**Fig. 1:**
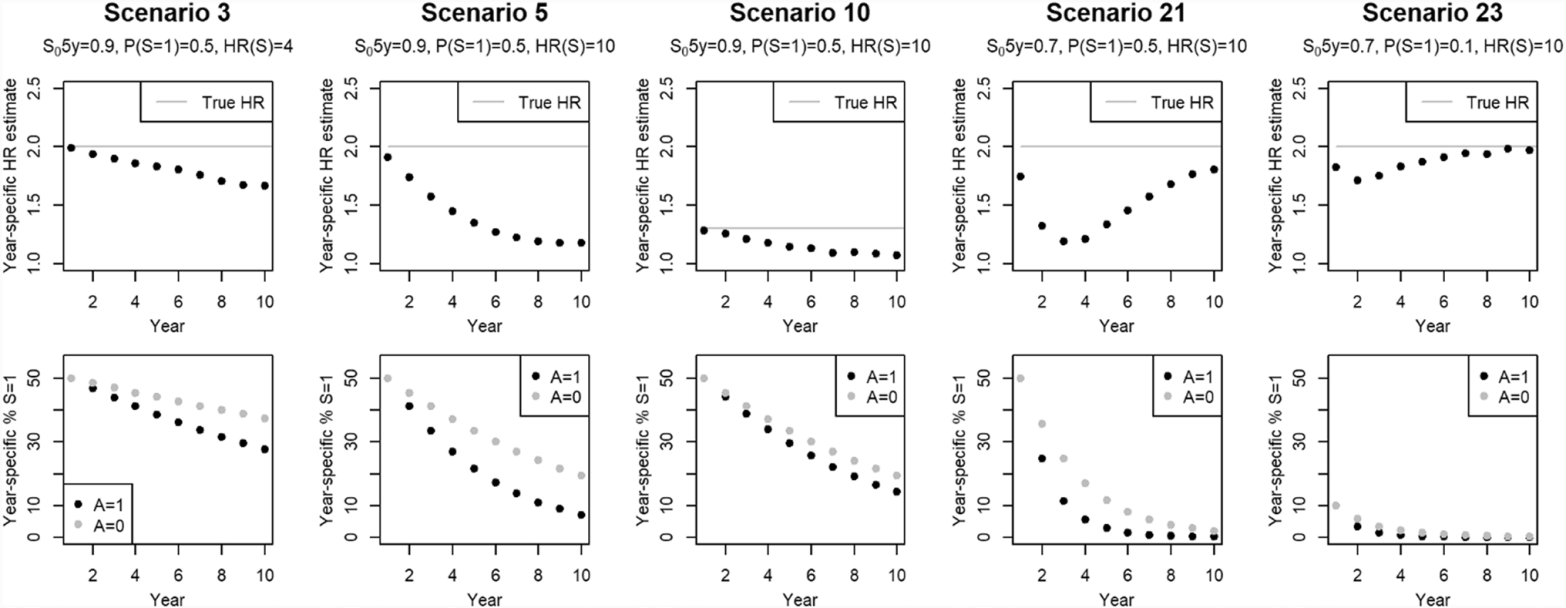
Changes over follow-up time in (i) bias of year-specific hazard ratio (HR) estimates for treatment* A* (*top panels*), and (ii) proportions of susceptible participants (*S* = 1) by treatment group (*A* = 1 vs. *A* = 0) (*bottom panels*). Results for five scenarios from Table 1, all with *N* = 4,000

Figure 1 explores more in detail how the biases reported above occur because of *differential* rates of depletion of susceptible participants (*S* = 1) across the two treatment groups, which is the main focus of Hernán’s critique of “built-in selection bias” affecting HRs [1]. For five scenarios of Table 1, the upper row of Fig. 1 shows how the mean, across the 1,000 simulated samples, of year-specific HR estimates changes with increasing follow-up time. The corresponding panels in the lower row illustrate how the mean proportions of susceptible participants (*S* = 1) vary with increasing follow-up, separately for the two treatment groups. Because *S* = 1 is associated with a much higher hazard, susceptible participants tend to have earlier events and their proportion P(*S* = 1|*t*) gradually decreases with time, in both groups. However, filtering out of susceptible persons is faster among treated (*A* = 1), who have a higher hazard, i.e. more frequent early events. Therefore, the discrepancy between the corresponding year-specific proportions P(*S* = 1|*A* = 0, *t*) versus P(*S* = 1|*A* = 1, *t*) increases with increasing year *t* (lower row of Fig. 1), at least until P(*S* = 1|*t*) approaches 0 for one or both treatment groups (Fig. 1). In summary, *S* (i) is not measured, (ii) impacts the hazard very strongly, and, (iii) with increasing follow-up time, becomes increasingly more prevalent in the non-treated group (*A* = 0). The combination of (i)-(iii) implies that, over time, *S* starts acting as an unmeasured confounder of year-specific HRs for treatment, even if randomization balances the distribution of *S* at time 0. Thus, a gradually increasing attenuation of the estimated treatment effects is due to a lower proportion of high-risk susceptible participants in the treated group (*A* = 1). Furthermore, the resulting changes in year-specific HRs (upper row of Fig. 1) imply a violation of the proportional hazards assumption.

On the other hand, Fig. 1 indicates also that year-specific HRs for treatment may sometime change in a *non-monotone* fashion, with early decreases followed by late increases. This is especially evident for scenario 21 where almost 80% of participants had events during the 10-year follow-up (Table 1, column 7). Because of their highly increased hazard (HR(*S*) = 10), most susceptible participants had events by year 6, when their proportions decreased to below 10% in both treatment groups. Accordingly, the discrepancy between P(*S*(*t*) = 1|*A* = 0) *vs.* P(*S*(*t*) = 1|*A* = 1) starts decreasing after year 5, and by year 10 both proportions shrank to almost 0 (Fig. 1, lower panel for scenario 21). Thus, by year 10, *S* was *not* associated with treatment anymore, resulting in an almost unbiased treatment year-specific estimate (Fig. 1, upper panel for scenario 21). Similarly, in scenario 23 the combination of high incidence with only 10% prevalence of susceptible resulted in very few susceptible participants remaining in either group in years 6–10, when the year-specific HRs are practically unbiased (Fig. 1). Interestingly, while early on bias is higher for scenario 5 with stronger treatment HR = 2 than for scenario 10 with a weak HR = 1.3, by year 10 the year-specific estimates for both scenarios show very similar relative biases (top panels of Fig. 1). Finally, for scenario 3 the bias in HR(*t*)’s is smaller (upper panel of Fig. 1) because the impact of important discrepancies between P(*S*(*t*) = 1|*A* = 0) *vs.* P(*S*(*t*) = 1|*A* = 1) is reduced by a relatively weaker HR(*S*) = 4.

In Table 1, scenarios 5, 10, and 11 combine (i) frequent *S* P(*S* = 1) = 0.5 with (ii) high impact HR(*S*) = 10 and (iii) high cumulative incidence, and their mean year-specific log(HR)’s decay to close to 0 by year 8 or 10. This raises the question to what extent the results of these scenarios may be seen as a solid evidence of the “crossing hazards” phenomenon discussed by Hernán [1]. Supplementary Material section S3 outlines additional simulations, carried out to explore this issue, and Supplementary Table S3 provides their detailed results. Interestingly, for the late year *t* = 10, 23%−38% or 6–30% of simulated samples, for *N* = 4,000 or *N* = 16,000 respectively, yield *negative* point estimates of year-specific log(HR)’s, with higher proportions for the weakest treatment HR(*A*) = 1.3 (scenarios 10 and S11 in Supplementary Table S3). Yet, especially for smaller samples, these results partly reflect just the sampling error. Indeed, for the lower *N* = 4,000, even in scenario S13 with a weak HR(*A*) = 1.3 but *no* unmeasured susceptibility, for any year *t*, at least 10% of year-specific log(HR(*A*)) estimates are negative. The importance of accounting for the impact of sampling error on year-specific estimates is underscored by our finding that in only < 2% of samples, the corresponding 95% CI excludes 0 for smaller *N* = 4,000 and this proportion falls further to < 1% for the larger *N* = 16,000 (Supplementary Table S3). Last not least, whereas up to 30%−40% (for *N* = 16,000 or 4,000) of year-specific estimates, for a given year, may be negative, only 2.5% or 4.0% of samples, respectively, yielded negative point estimates *consistently* for all three last years (*t* = 8–10) (last column of Supplementary Table S3). Yet, to report “crossing hazards”, one should observe the log(HR) estimates being *systematically* on the opposite sides of 0 for earlier *versus* late phases of follow-up, as illustrated e.g. by our real-world results in Fig. 4b and 4 d below. Overall, the detailed results in Supplementary Table S3 indicate that to reliably assess if the data truly support crossing hazards, investigators should consider not only year-specific point estimates but also their confidence intervals. Using such in-depth approach, we conclude that even a combination of a very strong susceptibility with HR(*S*) = 10 and a weak treatment effect (HR = 1.3 or 1.5) is very unlikely to induce a robust evidence of crossing hazards that is entirely due to unmeasured susceptibility, in the absence of a true time-dependent effect.

In summary, our simulations confirm that, as known from the literature, a strong unmeasured susceptibility *S* induces a differential depletion of susceptible participants during follow-up. This leads to time-dependent changes in year-specific HRs, which usually but not always decrease with time, inducing a violation of the PH assumption and bias toward the null in the overall estimates from the Cox PH model. However, the bias is generally small unless *S* is very strongly associated with the outcome, and is a complex function of the joint effect of the (i) susceptibility prevalence and (ii) cumulative incidence of the event during follow-up. In particular, if *S* is relatively rare and/or the cumulative incidence is high, the bias of year-specific estimates may start to decrease later during follow-up, after almost all susceptible participants were filtered out of the risk set, due to their earlier events.

### Results for continuous susceptibility

Table 2 summarizes the results of simulation scenarios where unmeasured susceptibility *S* is a continuous normally distributed N(0, 1) variable, with a linear relationship with the log hazard. In general, the pattern of results is similar to those in Table 1 for a binary *S*, with a few differences noted below. The overall Cox model-based log(HR)’s for treatment are systematically biased toward the null, with bias clearly increasing with stronger impact of *S* on the hazard. (True HR(*S*) of 2.0 to 3.0 for 1 standard deviation (SD) increase in *S* imply HRs of 4.0 to 9.0 for participants with *S* values 1 SD above *vs.* 1 SD below the mean, and HRs of 16 to 81 for 2 SDs above *vs.* 2 SDs below the mean.) Similar to binary *S* (Subsect."Results for binary susceptibility"), the bias in treatment effect is substantial only with large HR of 2.0 and above (for 1 SD increase in *S*). With more moderate HR(*S*) = 1.5 or 1.2, the bias is only minimal, below 7% or 13% for the overall or all year-specific estimates, respectively, (scenarios 30 and 31). In addition, as for binary *S*, for the same HR(*S*), the underestimation bias increases with lower baseline survival, i.e. with higher incidence of the event during follow-up (e.g., scenarios 29 vs. 34 vs. 37). In fact, if only less than 15% of the participants have events during follow-up, as often reported in real-world studies, the relative bias does not exceed 10% for the overall log(HR) and 15% for any of year-specific estimates, even for an extremely strong *S* with HR = 3.0 for 1 SD (scenarios 35–37). Furthermore, in comparison with results for a binary *S* in Table 1, for a continuous *S* biases are generally smaller and, even for year-specific estimates, never exceed 47% relative bias (Table 2). Another noticeable difference is that for a continuous *S*, bias in year-specific estimates increases *monotonically* with longer follow-up (Table 2). Indeed, the late decreases in bias seen for a binary *S*, especially with low prevalence (Table 1), occurred after almost all participants with the high-risk value *S* = 1 already had events (see Subsect."Results for binary susceptibility"and Fig. 1 for explanations), which cannot occur for a continuous *S*.

**Table 2:** Results of simulations with a continuous susceptibility *S*

Scenario	*N*	5-year baseline survival	True HR(S) for 1 SD increase in *S*	True HR(*A*)	Mean cumulative incidence, %	Overall relative bias, %	Year-specific (year 2) relative bias, %	Year-specific (year 4) relative bias, %	Year-specific (year 6) relative bias, %	Year-specific (year 8) relative bias, %	Year-specific (year 10) relative bias, %
27	4,000	0.7	3.0	2.0	58.7	−29.0	−23.8	−33.8	−39.6	−41.9	−46.6
28	4,000	0.7	2.5	2.0	59.0	−23.1	−16.5	−26.3	−31.6	−36.0	−39.4
29	4,000	0.7	2.0	2.0	59.3	−15.5	−9.6	−16.3	−21.9	−26.1	−27.9
30	4,000	0.7	1.5	2.0	59.6	−6.4	−2.9	−6.5	−8.3	−10.2	−12.5
31	4,000	0.7	1.2	2.0	59.7	−1.6	−1.0	−1.3	−2.0	−2.8	−2.2
32	4,000	0.9	3.0	2.0	31.3	−19.6	−11.8	−20.2	−23.8	−27.2	−30.4
33	4,000	0.9	2.5	2.0	30.0	−14.3	−7.5	−13.3	−17.7	−21.3	−22.6
34	4,000	0.9	2.0	2.0	28.3	−8.4	−3.8	−7.6	−8.3	−11.8	−14.0
35	4,000	0.97	3.0	2.0	12.7	−10.0	−3.7	−6.8	−8.1	−15.0	−14.1
36	4,000	0.97	2.5	2.0	11.4	−6.9	−1.2	−5.4	−6.4	−10.5	−7.7
37	4,000	0.97	2.0	2.0	10.1	−3.8	1.5	−1.5	−0.7	−4.4	−4.6
38	4,000	0.7	3.0	1.5	55.3	−27.8	−21.8	−32.2	−37.1	−40.8	−42.3
39	4,000	0.7	2.5	1.5	55.4	−21.4	−16.4	−22.3	−30.0	−33.6	−34.8
40	4,000	0.7	2.0	1.5	55.3	−14.7	−7.1	−15.3	−20.2	−24.0	−26.4
41	4,000	0.7	3.0	1.3	53.6	−26.8	−19.3	−28.8	−36.0	−39.7	−44.4
42	4,000	0.7	2.5	1.3	53.5	−21.8	−14.7	−23.7	−28.6	−35.3	−32.7
43	4,000	0.7	2.0	1.3	53.3	−13.8	−10.0	−14.7	−21.6	−21.0	−22.9

*HR* hazard ratio, *SD* standard deviation

In Supplementary Fig. S2, for selected scenarios, we illustrate how the bias in the HR estimate is related to faster rates of decrease over time in the mean value of *S* among participants in the higher-risk treated group (*A* = 1). The patterns are broadly similar to those seen in Fig. 1 for binary *S*, with further explanations provided in the Supplementary Material section S4.

## Targeted simulations to mimic the RCT discussed by Hernán

### Aims, design and assumptions

Below, we describe further simulations that aim to explore if, to what extent, and under what assumptions the empirical results of the RCT used as the main example in Hernán’s 2010 commentary [1] may be due to an unmeasured susceptibility. Accordingly, the new simulations were designed specifically to mimic the hormone therapy RCT by Manson et al. [7], later referred to as the “target trial”. Specifically, across simulations, we fixed (i) the sample size to the target trial’s *N* = 16,600, and (ii) true HR for treatment to 1.81, corresponding to the target trial’s estimate for year 1, assumed to represent the true treatment effect by Hernán [1]. Furthermore, our simulations of Sect."Simulation results assessing the impact of unobserved susceptibility"above indicate that the incidence rate of events during follow-up also affects the bias due to an unmeasured susceptibility. Thus, in all simulations of Sect."Targeted simulations to mimic the RCT discussed by Hernán", (iii) we censored all participants after 335 events occurred, to match the total number of coronary heart disease (CHD) events in the target trial [7]. Therefore, the only assumptions that varied across the simulated scenarios concerned the two unknown factors: (iv) the distribution of the unmeasured susceptibility *S*, and (v) its impact on the hazard (true ${\beta }_{s}$ in Eq. (1)). However, we considered only very strong effects of *S*, which are necessary to observe important biases, as shown in Tables 1 and 2. Finally, (vi) the mean number of events simulated in the last period of follow-up was about 64–67, which approximately matched the 65 CHD events in the corresponding period in the target trial [7]. Section S5 of Supplementary Material describes in detail how this was achieved.

### Results of targeted simulations

We focused on two issues that are directly relevant for assessing if the target trial results were likely to reflect just the impact of an unmeasured susceptibility and were the main focus of Hernán’s critique [1]. First, we compared the estimated overall Cox model-based treatment HRs, based on all 335 events, *versus* the overall HR = 1.24 reported by Manson et al. [7]. Secondly, we compared the period-specific HRs for the last period of the simulated trial, based on about 65 events, *versus* the corresponding HR = 0.7 reported for the target trial [7].

**Fig. 2:**
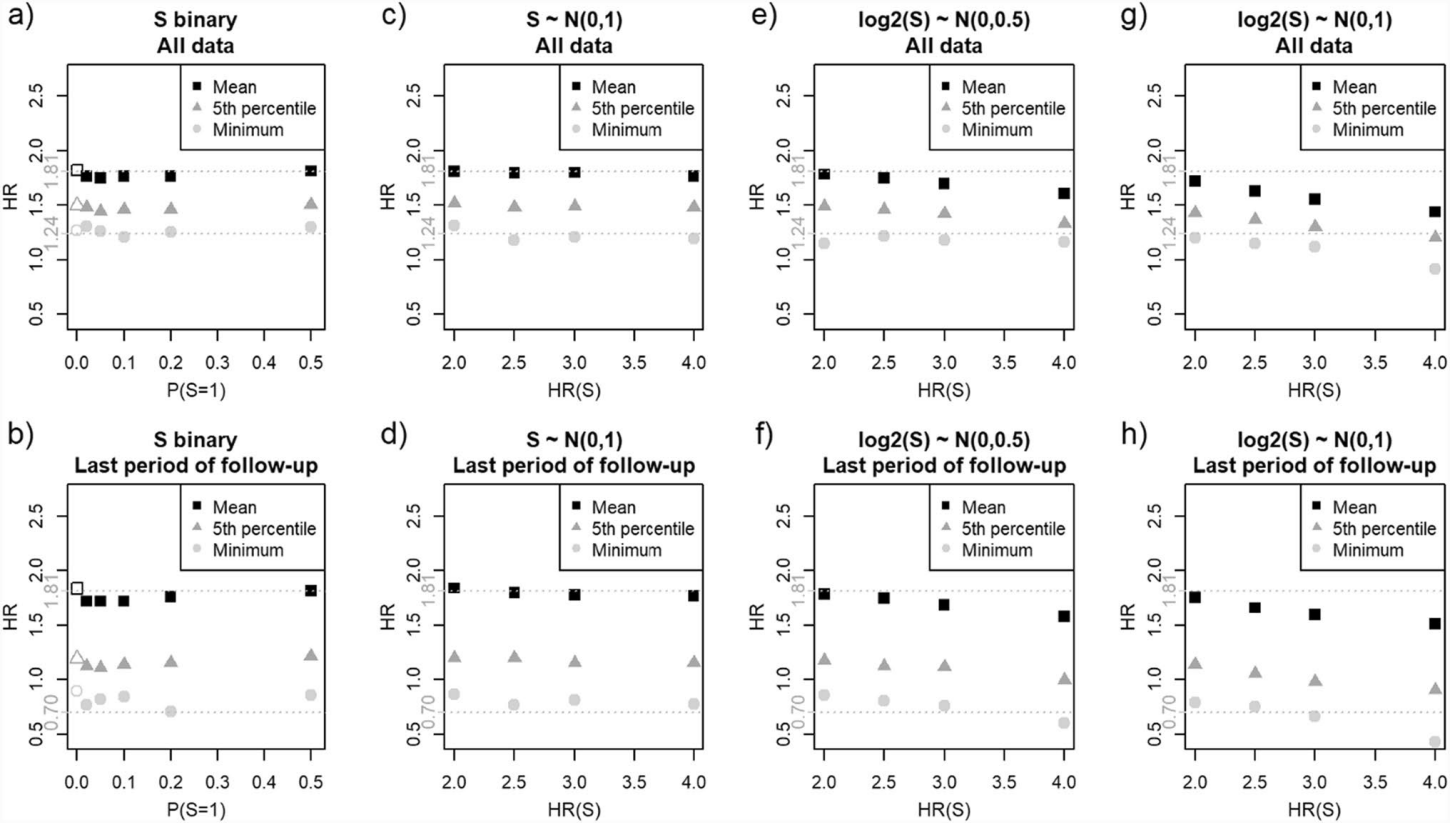
Comparing simulation-based estimates for treatment vs. hazard ratios (HRs) reported in the target trial for the overall Cox model-based HR (*upper panels*: **a**, **c**, **e**, **g**) and period-specific HR for the last period (*lower panels*: **b**, **d**, **f**, **h**). The reported HRs are indicated by lower horizontal dotted lines at 1.24 for the overall and 0.7 for last period-specific HR. The upper horizontal dotted line at HR = 1.81 indicates the true treatment HR used to simulate the data, corresponding to the 1 st year estimate from the target trial. The x-axis identifies different simulated scenarios by either prevalence P(*S* = 1) of a binary *S* (panels **a** and **b**), or HR(*S*) for 1 standard deviation increase in a continuous *S* (other panels). For continuous *S*, the panel headings indicate their distribution: normal for panels **c** and **d**, or lognormal with either moderate (panels **e** and **f**) or high positive skewness (panels **g** and **h**). For each scenario, the distribution of 1,000 treatment HR estimates is summarized by showing, along the y-axis, their means (*squares*), 5th percentiles (*triangles*) and minima (*circles*). Leftmost results in panels **a** and **b** correspond to scenario with P(*S* = 1) = 0, i.e. absence of an unmeasured susceptibility

Figure 2 summarizes the main results of several simulated scenarios, with upper and lower panels focusing on, respectively, the overall HRs and the last period-specific HRs for treatment. In each panel, the x-axis identifies different scenarios, and the corresponding distribution of the 1,000 estimates is summarized by showing along the y-axis their means (squares), 5th percentiles (triangles) and minima (circles). Figures 2a-2b summarize the results for a binary susceptibility *S* with a very strong HR(*S*) = 25. As expected, given the results of Sect."Simulation results assessing the impact of unobserved susceptibility", the mean estimates are almost always slightly lower than the corresponding true HR (upper dotted line), confirming a systematic, albeit small, bias toward the null. However, regardless of the prevalence P(*S* = 1), indicated on x-axis, not only the mean estimates (squares) but even the 5th percentiles (triangles) are always markedly higher than the corresponding HRs reported in the target trial (lower dotted lines): 1.24 for the overall HR (Fig. 2a) and 0.7 for the last period-specific HR (Fig. 2b). In fact, a value as low as the corresponding target trial estimate was found only in a *single* simulated sample for the overall HR with P(*S* = 1) = 0.1, and never for the last period-specific HR. Thus, even assuming an extremely strong impact of a binary unmeasured susceptibility, with HR(*S*) = 25, in only one of the 6,000 simulated samples we could recover the overall HR reported for the target trial [7]. Furthermore, the corresponding percentiles of the estimates for scenarios with P(*S* = 1) > 0 and very strong *S* effect were quite similar to those found for the scenario where *S* was absent, with P(*S* = 1) = 0 (leftmost scenario in Fig. 2a-2b). Yet, when P(*S* = 1) = 0, the low estimates *cannot* be attributed to susceptibility and are simply due to sampling variance. Thus, numerical instability of HRs based on only a few hundred events, or even less for period-specific HRs in Fig. 2b, can explain alone why a few samples may yield relatively low estimates, regardless of whether a strong susceptibility is absent or present.

Figures 2c-h show similar results for continuous *S* with three different distributions with increasing right skewness (different panels) and true HR(*S*) per 1 SD gradually increasing in each panel. As for a binary *S*, the mean estimates are reasonably close to treatment true HR = 1.81, but bias increases with stronger impact of *S*. For almost all scenarios only a very small percentage (not exceeding 1.5%) of simulated samples yielded an overall HR estimate as low as 1.24, reported for the target trial. The only scenario where this percentage increases markedly, to 9.6%, combines (i) an extremely strong impact of *S* with (ii) its highly skewed distribution (Fig. 2g). Regarding (i), the HR = 4.0 per 1 SD increase in *S* implies HRs of either 16 or 256 for comparing participants with *S* values either 1 SD or 2 SD above vs. either 1 SD or 2 SD below the mean, respectively. For the last period-specific HR, in most scenarios even the minima of the 1,000 estimates are above the target trial estimate of 0.7 (Fig. 2d, 2f and 2 h). In the most extreme scenario (rightmost part of Fig. 2h), only 0.5% of 1,000 samples had HR estimates below 0.7. Thus, for a continuous unmeasured susceptibility, it is extremely unlikely to observe the last phase period-specific treatment estimate as low as 0.7 reported by Manson et al. [7]. Furthermore, for the overall treatment effect, overall HRs as low as the target trial’s HR = 1.24 occur only under extreme assumptions of susceptibility *S*’s impact on the hazard, with a non-trivial, but low, probability.

In conclusion, given that, in our targeted simulations, the sample size, number of incident events, and true treatment HR all resembled closely the Manson et al.’s trial [7], the above findings indicate that the reported values of both overall HR = 1.24 and last period-specific HR = 0.7 are very unlikely to reflect exclusively the impact of unmeasured susceptibility. Accordingly, one should consider *alternative* reasons for the results of the target trial highlighted by Hernán [1].

### Alternative explanations for decaying treatment effect estimates

**Fig. 3:**
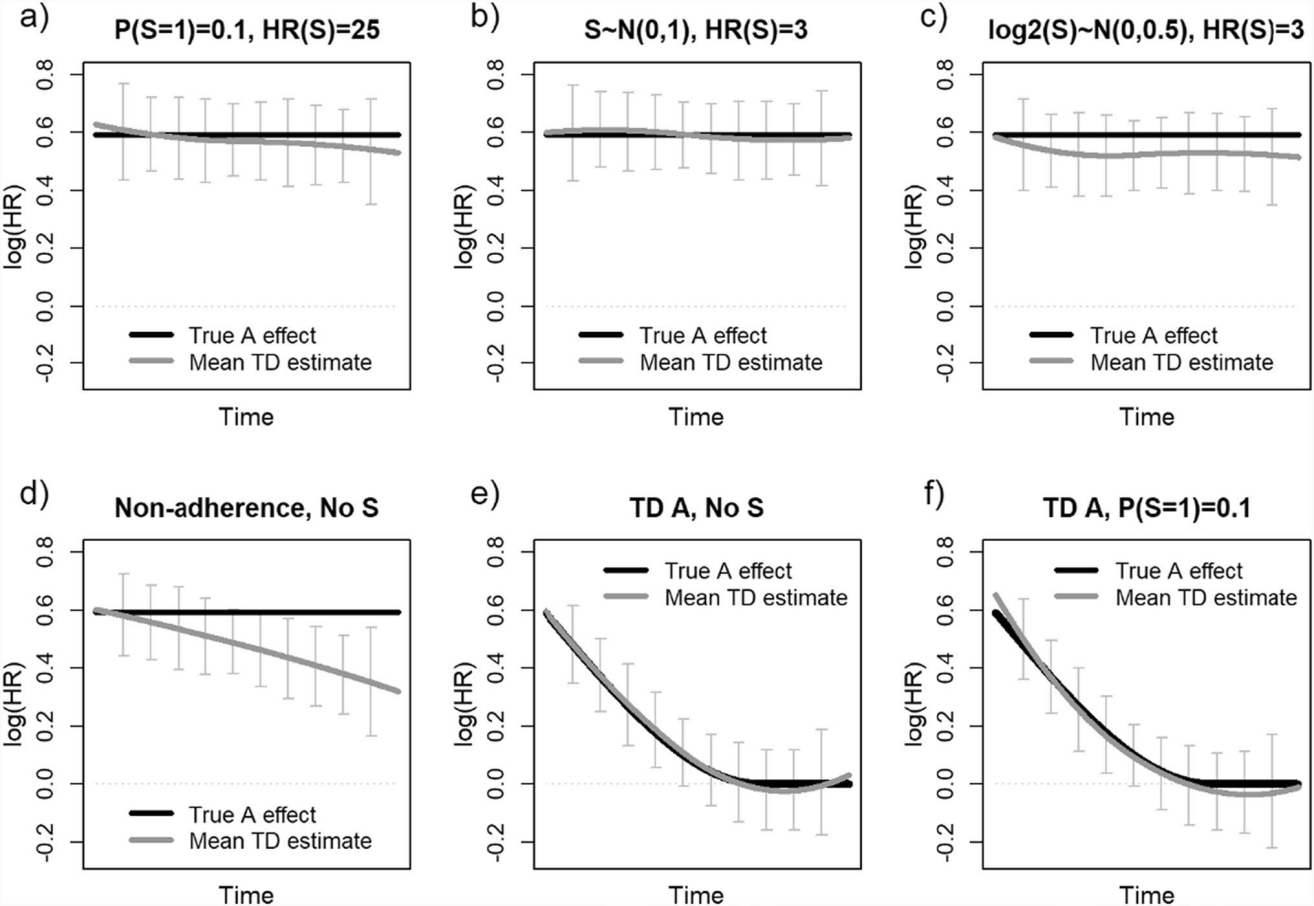
Spline-based time-dependent (TD) treatment effect estimates for selected scenarios of the targeted simulations reported in Subsect."Results of targeted simulations"(panels **a**−**c**), for treatment non-adherence of Subsect."Alternative explanations for decaying treatment effect estimates"(panel **d**), and for two scenarios with the true hazard ratio (HR) for the treatment gradually decreasing over time, either in the absence or in the presence of *S* (panels **e** and **f**, respectively). The distribution of susceptibility *S* and its true HR are indicated for the corresponding scenario (except for panels **d** and **e**). In each panel, the black curve indicates the true log HR (TD in panels **e** and **f**) for treatment *A*, and the dark grey curve the corresponding mean estimate across the 1,000 samples. The vertical bars at selected follow-up times *t* show the interquartile range (Q1-Q3) of the 1,000 TD log(HR) estimates corresponding to a given time *t*

Gray curves in Fig. 3a-3c show the means of spline-based estimates of time-dependent (TD) treatment effects for selected scenarios of targeted simulations, with sample size and incidence identical to the Manson et al.’s RCT [7] (headings of each panel in Fig. 3 identify the assumptions for the corresponding scenario). In all three scenarios there is no evidence of any systematic changes in the treatment log(HR), in spite of a very strong impact of *S*. Looking for other plausible explanations for the results of that RCT, we note that Manson et al. reported that 42% of women randomized to hormone therapy stopped using the treatment during follow-up [7].

To explore to what extent increasing treatment non-adherence could partly explain the observed changes in treatment HRs in the target trial, we modified two aspects of our targeted simulations described above in Subsect."Aims, design and assumptions". Specifically, we (i) eliminated the unmeasured susceptibility, but (ii) assumed that, among treated participants, 6% stop using the treatment in each year of the trial (consistent with the reported 42% non-adherence by seven years [7]). Thus, for the non-adherent women in the treatment arm, their true exposure is time-dependent, switching from *A*(*t*) = 1 to 0 at the time of treatment cessation. Finally, we assumed that hazard at time *t* depended on the current exposure *A*(*t*). We then used the permutational algorithm, specifically validated for simulating event times conditional on time-dependent exposures [16, 43–45], to generate simulated samples. This approach was validated as the Cox model with time-dependent current treatment *A*(*t*) yielded unbiased results (mean log(HR) = 0.596 vs. true 0.593).

Figure 3d shows that in simulations that assume non-adherence similar to that reported by Manson et al. [7], the mean TD estimate of log(HR) for baseline treatment (*A*, assigned at randomization) decreases much more clearly than in simulations with a very strong unmeasured susceptibility (Fig. 3a-3c). These simulation results suggest that estimates found in the hormone therapy trial [7] are likely more affected by increasing treatment non-adherence than unmeasured susceptibility. However, non-adherence cannot *fully* explain the results of the target trial. Indeed, although *not* mentioned by Hernán [1], the secondary per protocol analyses, reported in the original manuscript, limited to fully adherent women yielded overall Cox model-based HR = 1.50 (95% confidence interval: 1.14–1.97), considerably lower than the 1 st year HR = 1.81 [7].

To explore other reasons for the treatment HR decreasing over time, Fig. 3e and 3f present the time-dependent estimates of treatment effect for two *hypothetical* scenarios, in which the true harmful impact of *A* gradually decreases from the initial log[HR(*A*)] = 1.81 to null log[HR(*A*)] = 0 in the second half of follow-up. The mean estimates (gray curves) recover this pattern very well with no marked bias even in the presence of a strong unmeasured susceptibility (Fig. 3f). In conclusion, results of our targeted simulations call for a careful consideration of possible biological reasons for time-dependent treatment effect in the hormone therapy trial. Together, all simulation results presented in this section suggest that there likely is another reason, beyond unmeasured susceptibility, on which Hernán focuses [1], acting in addition to increasing non-adherence, why the harmful effect of hormone therapy on CHD risks gradually decreases with increasing trial duration.

Interestingly, Stensrud et al. [46] used simulations in an attempt to show that the estimates found in the Mason et al.’s trial [7] might be due to unmeasured susceptibility. However, in Supplementary Material section S6 we demonstrate that their simulations relied on more extreme and clinically implausible assumptions.

## Time-dependent hazard ratios for real-world cancer studies

### Overview of goals and methods

Our targeted simulations suggest that the decrease in adverse CHD effects of hormone therapy, with increasing treatment duration, may be possibly due to some inherent properties of the treatment, rather than to unmeasured susceptibility. Whereas we cannot identify the specific mechanism underlying such potential truly time-dependent treatment effect in the Manson et al.’s trial [7], to support our conjecture, below we provide real-world examples of clinically plausible and interpretable time-dependent HRs in cancer studies. We used the penalized likelihood approach described in Luo et al. [47] to fit a flexible extension of the multivariable Cox model with ${\beta }_{X}(t)$ modeling the time-varying changes in the adjusted log(HR) for covariate *X*.

### Increasing-over-time benefits of a novel immunotherapy in lung cancer

**Fig. 4:**
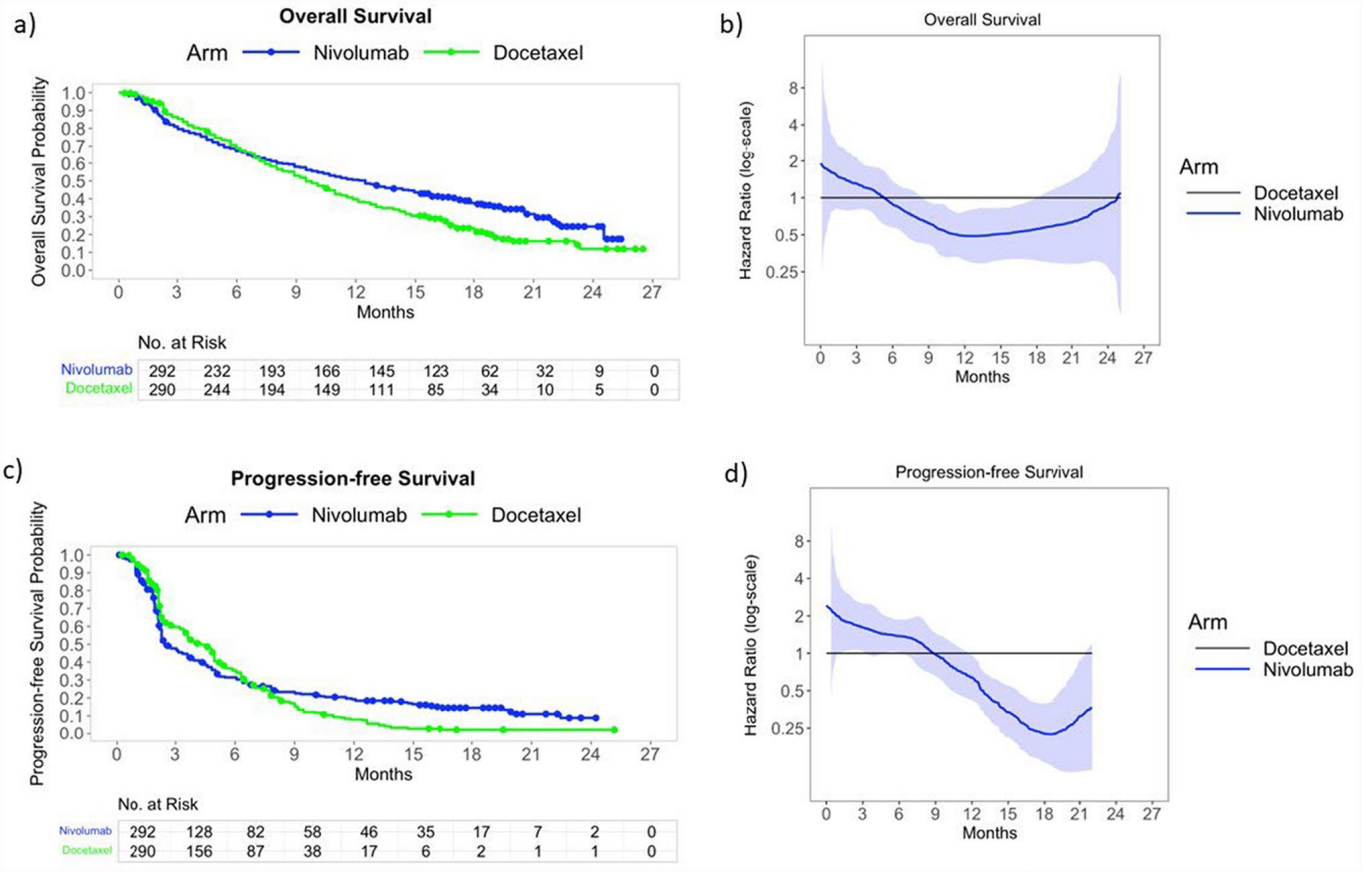
Results for overall (* 1 st row*) and progression-free (*2nd row*) survival from reconstructed data of the Checkmate 057 randomized trial: panels **a** and **c** show Kaplan–Meier estimates for immunotherapy with nivolumab *versus* docetaxel; panels **b** and **d** show time-dependent hazard ratio estimates for nivolumab

The first example uses reconstructed data from the CheckMate 057 RCT of immunotherapy for 582 lung cancer patients [48]. Figure 4a compares overall survival for nivolumab, a new agent, *versus* docetaxel, the standard chemotherapy at that time. The PH assumption is clearly violated, with nivolumab showing a slight hazard increase in the first few months, but a *delayed* beneficial effect apparent only after 6 months of treatment. A similar pattern has been seen in other immunotherapy trials. The early months of the corresponding time-dependent HR graph (Fig. 4b) reflect the immediate effect of docetaxel, designed to shrink the tumor or slow its growth, likely preventing some early deaths. The mechanism of action of nivolumab is not expected to have such an immediate effect. In contrast, the later years likely reflect a clinically plausible *cumulative* treatment effect: nivolumab must be taken regularly over several months to help the immune system gradually suppress the cancer growth. Importantly, such time-dependent effect, with treatment benefits *increasing* later in follow-up, cannot be due to an unmeasured susceptibility which induces gradual decay toward the null, as demonstrated in simulations of Sect."Simulation results assessing the impact of unobserved susceptibility". Figure 4c compares progression free survival (PFS) for nivolumab versus docetaxel, and Fig. 4d shows the corresponding time-dependent HR estimates. The PH assumption is clearly violated as the survival curves cross. PFS is determined by cancer growth or spread, based on images taken at regular intervals. Yet the immunotherapy may cause short-term inflammation, sometimes mistaken on the image for tumor progression. This “false progression” may explain why the PFS looks initially worse for the nivolumab group. In conclusion, this RCT illustrates clinically plausible time-dependent treatment effects that cannot be attributed to an unmeasured susceptibility and need to be considered to avoid underestimating longer-term benefits of nivolumab. This provides strong empirical evidence against the generalization of Hernán’s arguments and, in addition, illustrates the importance of assessing time-dependent effects, in contrast to recent recommendations against their testing and reporting [35].

### Prognostic factors for mortality in head and neck cancer

**Fig. 5:**
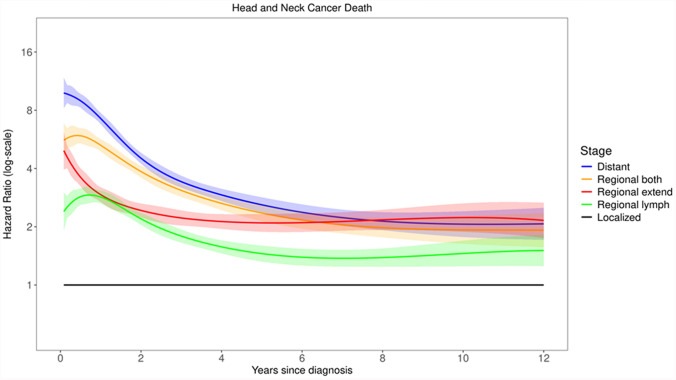
Time-dependent hazard ratio estimates for different stage groups, relative to localized disease, for their associations with the hazard of cancer-related mortality, among persons diagnosed with head and neck cancer between 2004 and 2017 in the SEER (Surveillance, Epidemiology, and End Results) registries

The second real-word example focuses on the association of cancer stage at diagnosis with cancer mortality among 107,954 people diagnosed with head and neck cancer between 2004 and 2017 in the SEER (Surveillance, Epidemiology, and End Results) registries [24, 47]. The analyses adjusted for age, sex, race, year of diagnosis, histology, grade and subsite. We focus on cause-specific hazards for the 36,374 deaths due to head and neck cancer, while censoring the 21,963 deaths due to other causes. Figure 5 shows the adjusted time-dependent HRs comparing different cancer stage at diagnosis *versus* the most benign stage (localized disease) for cancer-related mortality in SEER cohort [24, 47]. For the most advanced stage (distant metastasis), the adjusted HR decreases greatly from about 8 in the first year after diagnosis to 2 six years later. These changes are similar to estimates from other studies [20, 24, 47], and are clearly interpretable. Initial mortality is largely driven by cancer stage at diagnosis. Most patients are treated with curative intent, which removes the cancer or arrests its growth. This greatly reduces the risk of cancer recurrence, which for head and neck cancer occurs mostly in the first 5 years. If a patient survives beyond 5 years, their *initial* cancer stage becomes considerably less relevant than it was soon after diagnosis.

Supplementary Material section S7 reports further SEER analyses focusing on the association between the hazard of cancer-related mortality and age at diagnosis, which also show marked, clinically interpretable time-varying effects. Importantly, for the same outcome, the patterns of estimated changes for age (Supplementary Figure S3) *versus* stage (Fig. 5 above) are quite different, indicating they *cannot* reflect the impact of the same unmeasured susceptibility.

## Additional simulations based on real-world studies with time-dependent exposure effects

### Aims

In Sect."Time-dependent hazard ratios for real-world cancer studies"we have provided both (i) substantive reasons why the estimated time-dependent (TD) effects of prognostic factors or treatment are clinically plausible, and (ii) empirical evidence and formal arguments suggesting that these estimates are very unlikely to reflect just an unmeasured susceptibility. However, we cannot know whether the true effects of these real-world exposures are really constant or time-dependent, nor if some strong unmeasured predictors affect the results. For these reasons, below we report additional simulations designed specifically to objectively assess if real-world results can help discriminate between truly TD exposure HRs *versus* spurious changes over time in HR estimates induced by a strong unmeasured susceptibility. To ensure clinical plausibility of the simulations and, thus, enhance the practical relevance of results, we considered two different simulation series, each based on an impactful real-world study that reported a clinically plausible statistically significant TD exposure effect [19, 27].

### Overview of methods

The two additional series of simulations reproduced the following salient characteristics of their respective real-world study [19, 27]: the (i) frequency of the binary exposure (*A*) of interest, (ii) follow-up duration, and (iii) cumulative incidence rate of events. Further details are provided below in Subsect."Results and conclusion". Each simulation series involved three different scenarios. In the 1 st scenario, the hazard depended only on the exposure, with a *true* TD HR corresponding to the estimate from the respective real-world study. In the 2nd and 3rd scenarios, the hazard was affected by both the exposure *A* and a strong binary susceptibility *S* with true HR(*S*) = 10, independent of exposure *A* and generated from a binomial distribution with P(*S* = 1) = 0.5. In the 2nd scenario, the exposure had the same true TD effect as in the 1 st scenario, whereas in the 3rd scenario, the exposure true HR was constant over time and equal to the respective real-world estimate corresponding to the first month of follow-up.

Event times were generated from the exponential distribution, with a constant hazard rate defined to get the pre-specified incidence rate during follow-up. Then, we employed the permutational algorithm [16, 43–45] (see Subsect."Alternative explanations for decaying treatment effect estimates") to assign each event time to a participant, in accordance with the corresponding true model of the respective scenario, described in the previous paragraph. All participants not assigned any event in a given simulated sample were administratively censored at the end of follow-up.

Simulated data were analyzed using a flexible spline-based extension of the Cox model to estimate a TD log(HR)(*t*) for exposure [41, 42], without adjusting for susceptibility *S*. The estimated curves were compared with the true TD exposure effect, and across the three scenarios. We also compared the percentage of simulated samples in which the model-based likelihood ratio test (LRT) [41, 42] rejected the PH hypothesis at *α* = 0.05.

### Results and conclusion

**Fig. 6:**
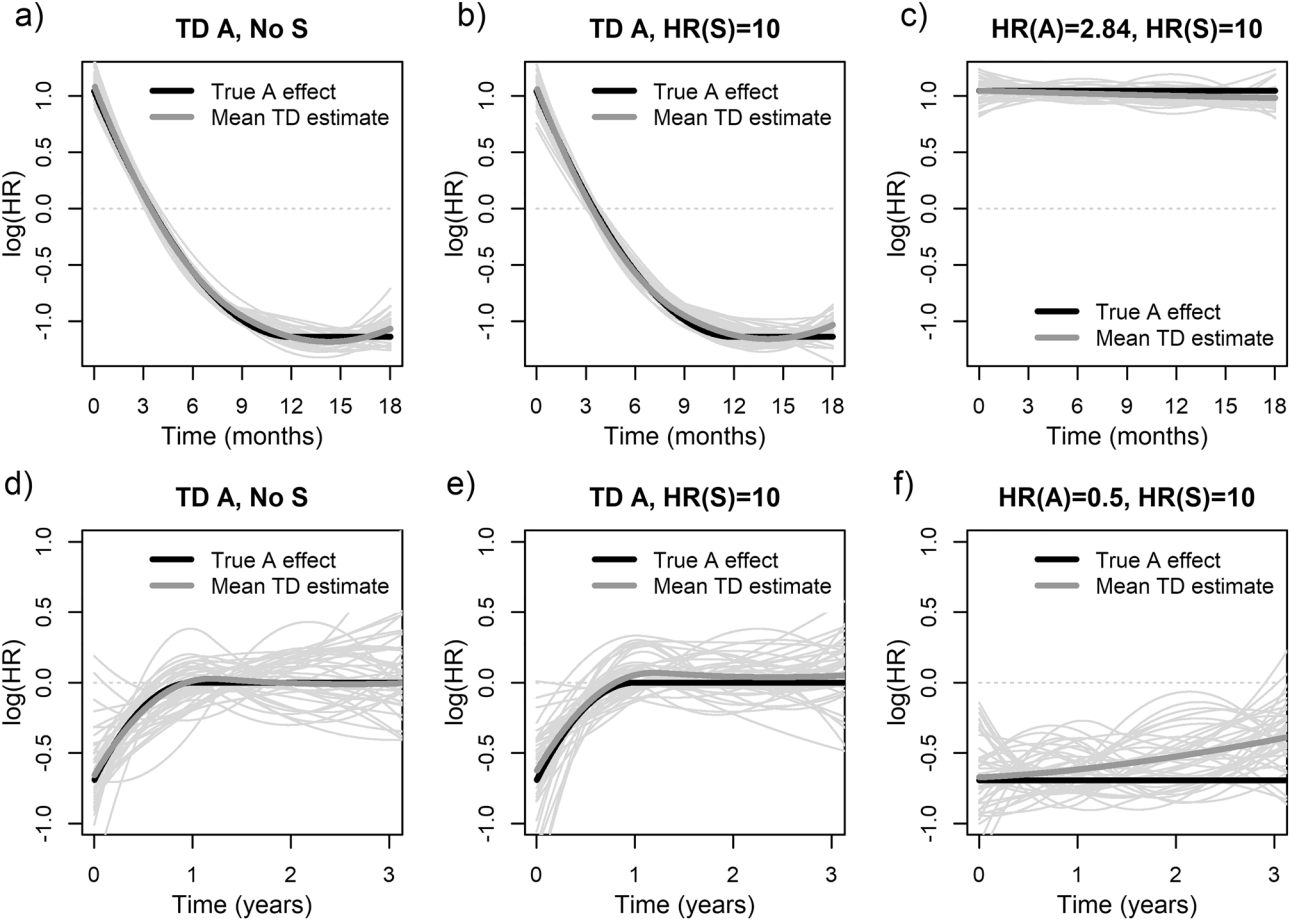
Spline-based time-dependent (TD) exposure effect estimates for simulations reported in Sect."Additional simulations based on real-word studies with time-dependent exposure effects". Panels **a**−**c** report results for the first simulation series mimicking a large cohort study of the effect of a cadaveric kidney transplant [27]. Panels **d**−**f** report results of the second simulation series, based on a randomized placebo-controlled trial of low-dose aspirin to prevent cardiovascular events in asymptomatic patients with carotid stenosis [19]. In each panel, the black curve indicates the true log hazard ratio (HR) for treatment *A*, and the thick dark grey curve the corresponding mean estimate across the 1,000 samples. The light grey curves are a random selection of 40 individual TD estimates

The first simulation series mimicked a highly cited large study by Wolfe et al. in the New England Journal of Medicine (> 6,600 citations on Google Scholar) of the impact of receiving a cadaveric kidney transplant on survival among *N* = 41,146 dialysis patients on the waiting list [27]. Replicating the reported results, in each simulated sample we generated about 4,340 deaths during a follow-up period of 1.5 year, and compared survival of 23,275 transplant patients *versus* 22,871 patients who remained on the waiting list [27]. In 1 st and 2nd simulated scenarios, the true transplant effect was assumed to match the TD effect reported in Fig. 2 of the original paper, with HR decreasing from 2.84 (i.e. log[HR] = 1.04) in the first month to 1.00 (log[HR] = 0) after 3.5 months and 0.32 (log[HR]=−1.14) at 12 months, after which it remained constant until the end of follow-up [27] (black curves in Fig. 6a and 6b). Top row of Fig. 6 summarizes the results of the three simulated scenarios. For both scenarios with the true TD effect of transplant, all estimated curves (thin grey curves) match closely the true TD HR, and are almost identical regardless of whether the strong susceptibility *S* was absent or present in the true model (Fig. 6a versus 6b, respectively). In the 3rd scenario, the true transplant effect was assumed constant with HR = 2.84 (log[HR] = 1.04) corresponding to the initial 1 st month estimate in the original publication [27]. Here, in spite of the presence of a very strong unaccounted for susceptibility (HR(*S*) = 10), the estimated curves are mostly flat (Fig. 6c), and very different from those obtained with a truly TD transplant effect. Furthermore, the proportion of simulated samples where the LRT rejected the PH assumption was 100% for both scenarios with truly TD transplant effect, but it decreased to only 8.7% for the 3rd scenario with constant true HR and strong susceptibility.

The second simulation series was based on a randomized placebo-controlled trial of low-dose aspirin to prevent cardiovascular events in asymptomatic patients with carotid stenosis [19]. To get stable estimates, the original sample size (*N* = 372) and number of events (104) were multiplied by five, to *N* = 1,860 patients (940 on aspirin, 920 on placebo), with 520 events. Patients were followed for up to four years, with a median of two years due to staggered entry into the original trial [19]. Similar to the original trial, the incidence rate was about 12 events per 100 person-years. To replicate the spline-based TD exposure estimate in Fig. 2 of the original publication [19], the 1 st and 2nd scenarios assumed the true TD effect of aspirin decayed from an early protective HR = 0.5 (i.e. log[HR] = −0.69) around *t* = 0, to stabilize at null log(HR) = 0 for all *t* ≥ 1 year. Again, the vast majority of TD estimates for both simulated scenarios recover well the decaying protective effect regardless of whether a strong unmeasured susceptibility was absent or present (Fig. 6d *vs.*  6e, respectively), with a similar power to reject the PH hypothesis in both scenarios (37.0% *vs.* 36.6%). In contrast, in the absence of a true TD effect, most estimates for the 3rd scenario are relatively flat, in spite of a strong unmeasured susceptibility (Fig. 6f), with only 12.4% samples showing a statistically significant TD effect at *α* = 0.05.

Overall, results in Fig. 6 clearly indicate that, especially in larger datasets, flexible extensions of the Cox model can reliably discriminate between (i) true time-dependent exposure effects *versus* (ii) artificial changes in the estimated HR induced by a strong unmeasured susceptibility. It is also important to emphasize that in both real-world examples considered, the estimated TD effects are clinically and biologically plausible. Indeed, Wolfe et al*.* explicitly state that initial risk increases, in a few weeks after a kidney transplant, whose long-term benefits are undeniable, are expected due to a combination of the direct impact of a major surgery and adverse effects of high-dose immunosuppressive therapy [27]. On the other hand, a decaying protective effect of low-dose aspirin found by Cote et al. [19] is consistent with both a meta-analysis of several similar trials [49] and a basic study suggesting quick development of resistance to aspirin administered at a constant dose [50].

## Discussion

Built-in selection bias and non-collapsibility of hazard ratios are often identified as inherent limitations of the PH model [37, 51, 52]. However, there is less clarity regarding the magnitude of the resulting bias. Our simulations of Sect."Simulation results assessing the impact of unobserved susceptibility"illustrate how the bias to the null, and the artificial changes in year-specific HR estimates, induced by an unmeasured susceptibility *S,* occur and how they depend on the impact of *S* on the hazard and on its distribution, as well as on the incidence rate of the events observed during follow-up. Our results indicate that the bias is important only if *S* is a very strong risk factor and the cumulative incidence is relatively high. Then, in Sect."Targeted simulations to mimic the RCT discussed by Hernán"we report targeted simulations designed specifically to mimic, in terms of true treatment HR, sample size and number of events, the trial assessing CHD risks associated with hormone therapy [7] used as the main example in Hernán’s influential commentary on hazards of hazard ratios [1]. We demonstrate that there is only a negligible probability that even a very strong *S* could induce either of the two most striking empirical results of the original trial: (i) large bias toward the null of the overall treatment HR estimate (1.24 vs. true 1.81), and (ii) the crossing hazards, with year-specific HR for the last period as low as 0.7 [7].

Some of our results in Sect."Simulation results assessing the impact of unobserved susceptibility", concerning the bias dependence on a combination of the prevalence of a binary susceptibility *S* and incidence of the events, confirm those of Fireman et al. [38], who focus mostly on the impact of *S* on propensity score adjusted treatment effect estimates. Despite the different focuses and methods, both Fireman et al. and our study conclude that conditional HRs, even if affected by unmeasured susceptibility, may yield practically useful and interpretable estimates [38].

The results of our simulations in Sect."Targeted simulations to mimic the RCT discussed by Hernán"complement a recent review of several major cardiovascular trials that *failed* to find evidence of built-in selection bias or unmeasured susceptibility [11]. Using very distinct approaches, both studies present solid empirical evidence that strongly suggests that the Manson et al.’s results highlighted by Hernán [1] are primarily due to a cause different than unmeasured susceptibility. Together, these empirical results suggest shifting attention from a hypothetical susceptibility which, to our knowledge, was *not* identified in other studies of cardiovascular outcomes, to the properties of the specific treatment. Indeed, our additional simulations, in Subsect."Alternative explanations for decaying treatment effect estimates", demonstrated that the gradual decay in the treatment impact over time may be partly due to increasing treatment non-adherence, which was reported by Manson et al. [7] but *not* considered by Hernán [1]. Furthermore, with increased treatment duration, women may become gradually less sensitive to adverse cardiovascular effects of the treatment, as reported for example for cardiovascular risks associated with some powerful treatments in persons living with HIV [29, 30]. Indeed, Prentice and Aragaki present time-varying re-analyses of the Manson et al.’s hormone therapy trial and suggest searching for the underlying biological cause for the treatment impact decaying over time [28]. By demonstrating that the reported decrease in treatment HR is very unlikely to reflect just an unmeasured susceptibility, our simulation results in Sect."Targeted simulations to mimic the RCT discussed by Hernán"support the need for such a search. In fact, the comprehensive review by Hodis and Mack [53] reports that a gradual improvement in the relative risks of cardiovascular events with long-term hormone therapy is found *consistently* in (i) major RCTs [7, 54], (ii) the large Nurses’ Health Study cohort [55], and (iii) case–control studies. These epidemiological findings are compatible with imaging studies of the coronary artery plaque in which the benefits show only after several years of hormone therapy [56, 57]. Finally, Hodis and Mack discuss potential biological reasons for dual effect of hormone therapy with early risk increases followed by benefits associated with long-term treatment. Whereas, of course, simulations *cannot* identify the true reason(s) for time-varying changes in the HR for a specific treatment, our simulations of Sect."Targeted simulations to mimic the RCT discussed by Hernán"are in line with the evidence summarized in Hodis and Mack’s review [53], by suggesting that the estimates reported by Manson et al. [7] likely reflect some *genuine* time-varying changes in the treatment impact. To provide further empirical support for this conclusion, in Sect."Time-dependent hazard ratios for real-world cancer studies"we present examples of such time-dependent hazard ratios in large real-world epidemiological studies of cancer mortality. Importantly, these time-dependent estimates are interpretable and clinically plausible, including e.g. increasing-over-time benefits of a new immunotherapy treatment in a randomized trial, which likely reflect *cumulative* effects of prolonged therapy. These results add to a large body of real-world examples of interpretable time-dependent effects of many risk/prognostic factors and exposures (e.g. [17–27]), including treatments evaluated in RCTs (e.g. [19, 22]). Finally, in Sect."Additional simulations based on real-world studies with time-dependent exposure effects"we report additional simulations, designed to closely mimic real-world studies that reported statistically significant, clinically interpretable time-dependent exposure effects. These additional simulation results provide strong evidence that such changes over time in exposure HRs could *not* reflect simply an unmeasured susceptibility, even with a very strong HR(*S*) = 10.

In summary, whereas we recognize that, in the presence of unmeasured susceptibility, the Cox model and its flexible time-varying extensions do not yield unbiased estimates of a causal treatment effect, our simulation results offer insights regarding both the strength of the resulting bias and its determinants. From this perspective, our study supports four major conclusions, with potentially important practical and methodological implications. First, our simulation results indicate that the resulting built-in selection bias is relatively small unless the unmeasured susceptibility has a very strong impact on the survival, and that even under extreme assumptions this bias is unlikely to reliably suggest crossing hazards. Secondly, for the specific trial highlighted by Hernán [1], our targeted simulations demonstrate that the reported changes in treatment HR over time are unlikely to primarily reflect the built-in selection bias due to unmeasured susceptibility. Furthermore, from a more general perspective, we illustrate how well-designed simulations can help exploring a complex analytical issue. Finally, we provide empirical evidence and methodological arguments in favor of extending conventional time-to-event analyses to include flexible modeling of possibly time-dependent effects of treatments and risk/prognostic factors, and interpreting their results based on substantive knowledge. We hope that our study will encourage more frequent use of both simulations and time-dependent effect modeling in epidemiological research.

## Supplementary Information

Below is the link to the electronic supplementary material.

Supplementary file1 (PDF 495 kb)
