# Supplementary material for: Revisiting the hazards of hazard ratios through simulations and case studies
Source: Eur J Epidemiol. 2025 Jul 3;40(6):611–29. doi: 10.1007/s10654-025-01245-6 (PMC12263769; doi:10.1007/s10654-025-01245-6)
Supplement: Supplementary file 1 — Supplementary file1 (PDF 495 kb) [file 10654_2025_1245_MOESM1_ESM.pdf]

Supplementary Material

Revisiting the Hazards of Hazard Ratios Through Simulations and Case Studies

European Journal of Epidemiology

Michal Abrahamowicz[1,2,*], Marie-Eve Beauchamp[2], Emily K Roberts[3], Jeremy M G Taylor[4]

[1] McGill University, Department of Epidemiology, Biostatistics and Occupational Health, Montreal, Quebec, Canada
[2] Research Institute of the McGill University Health Centre, Centre for Outcomes Research and Evaluation (CORE), Montreal, Quebec, Canada
[3] University of Iowa, Department of Biostatistics, Iowa City, IA, USA
[4] University of Michigan, Department of Biostatistics, Ann Arbor, MI, USA
* Corresponding author: Michal Abrahamowicz, michal.abrahamowicz@mcgill.ca

S1. Joint impact of the prevalence of susceptibility and event incidence on bias

Supplementary Fig. S1 helps assess how the relative bias of the in the overall log(HR) (β_A), from the Cox PH model fit to all data, for treatment *A* varies across combinations of (a) prevalence of susceptibility P(*S*=1) (column 4 of Table 1), and (b) baseline survival rate (column 3), which determines the cumulative incidence of events during follow-up (column 7). Interestingly, for prevalences of 0.1 and 0.25, the relative bias is a non-monotonic function of survival rate, with largest bias for the intermediate baseline survival of 0.9. In contrast, when the prevalence increases to 0.5, the highest bias corresponds to the lowest survival of 0.7 (circles in Supplementary Fig. S1). Bias is almost identical for survival rates of 0.7 and 0.97 for a low prevalence of *S* of 0.1, but when the prevalence increases to 0.5, the bias is more than twice times larger for the 0.7 (circles) than for the 0.97 (squares) baseline 5-year survival rate. Similarly, the impact of prevalence on bias depends on survival rates. For baseline survival rate of 0.7 or 0.9, the bias increases sharply with increasing prevalence (light and dark grey curves in Supplementary Fig. S1). In contrast, with high baseline survival rate of 0.97, the strongest bias corresponds to an intermediate prevalence P(*S*=1) = 0.25 (black curve).

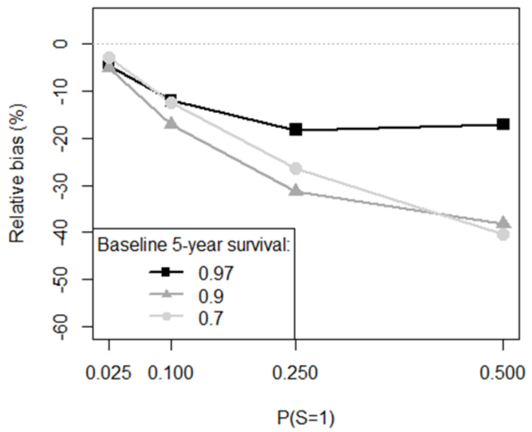

Supplementary Fig. S1 Joint impact of the susceptibility prevalence (P(*S*=1) on the x-axis) and baseline survival rate (different symbols) on the relative bias of Cox model-based log hazard ratio estimates for treatment *A.*

S2. Additional simulation results for highly prevalent binary susceptibility *S*

Supplementary Table S1 below shows results of some simulation scenarios in which a binary susceptibility *S* had a high prevalence P(*S*=1) > 0.5. As for the main simulations, summarized in Table 1 of the main manuscript, the bias in the overall log(HR) (β_A), from the Cox proportional hazards (PH) model fitted to all data, tends to generally decrease as the prevalence moves away from 50% (Supplementary Table S1). However, Supplementary Table S2 indicates a more complex dependence of the strength of the bias on a combination of the prevalence of *S* and cumulative incidence of events observed during follow-up. In each of these additional simulations, we compare the bias for two scenarios with P(*S*=1) of either 0.1 or 0.9, while fixing the observed incidence to a common level of either 35%, 65%, or 85% (paired scenarios shown with the same color). With lower incidence, more bias is observed when the prevalence is lower (Supplementary Table S2). In contrast, with high incidence of 85%, the bias is twice larger for P(*S*=1) = 0.9 than for lower prevalence of 0.1 (bottom of Supplementary Table S2). These complex results can be attributed to differential depletion of susceptible participants across the two treatment groups. Indeed, as illustrated (for different scenarios) in Fig. 1 of the main manuscript, both the extent of depletion and the pattern thereof during follow-up depend on the (i) prevalence of *S* and (ii) incidence of events during follow-up.

Supplementary Table S1 Results of simulations with a binary susceptibility *S* with (PS=1) > 0.5

Scenario	*N*	5-year baseline survival	P(*S*=1)	True β_S (HR)	True β_A (HR)	Mean cumulative incidence, %	Mean estimate β_A	Bias	Relative Bias, %	SD
S1	4,000	0.7	0.75	2.303 (10)	0.693 (2.0)	89.6	0.406	-0.287	-41.4	0.035
S2	4,000	0.7	0.9	2.303 (10)	0.693 (2.0)	95.6	0.475	-0.218	-31.4	0.036
S3	4,000	0.7	0.975	2.303 (10)	0.693 (2.0)	98.6	0.589	-0.104	-15.0	0.034

HR, hazard ratio; SD, standard deviation.

Supplementary Table S2 Results of additional simulations with a binary susceptibility *S* contrasting scenarios with P(S=1) = 0.1 vs. P(S=1) = 0.9 with the same incidence fixed to either 35%, 65%, or 85%

Scenario	*N*	Fixed incidence	P(*S*=1)	True β_S (HR)	True β_A (HR)	Mean cumulative incidence, %	Mean estimate β_A	Bias	Relative Bias, %	SD
S4	4,000	35%	0.1	2.303 (10)	0.693 (2.0)	35.0	0.572	-0.121	-17.4	0.056
S5	4,000	35%	0.9	2.303 (10)	0.693 (2.0)	35.0	0.671	-0.022	-3.2	0.056
S6	4,000	65%	0.1	2.303 (10)	0.693 (2.0)	65.0	0.608	-0.085	-12.3	0.041
S7	4,000	65%	0.9	2.303 (10)	0.693 (2.0)	65.0	0.631	-0.062	-9.0	0.040
S8	4,000	85%	0.1	2.303 (10)	0.693 (2.0)	85.0	0.627	-0.066	-9.5	0.035
S9	4,000	85%	0.9	2.303 (10)	0.693 (2.0)	85.0	0.547	-0.146	-21.1	0.036

HR, hazard ratio; SD, standard deviation.

S3. Additional simulation results for exploring "crossing hazards" with binary susceptibility *S*

Supplementary Table S3 shows additional results for scenarios 5, 10 and 11 of section 3.2 of the main manuscript, as well as new scenarios S10-S16. These results help evaluate whether, and to what extent, the year-specific log(HR)s for treatment might potentially drop below 0 during the later phase of follow-

up, even if the true log(HR) remains constant over time and above 0. Scenarios 5, 10, and 11 from Table 1 have been selected because they combine characteristics that could increase the probability of observing crossing hazards, i.e. (i) frequent *S* (P(*S*=1) = 0.5) with (ii) high impact HR(*S*)=10 and (iii) high cumulative incidence. Indeed, for these scenarios, the corresponding mean year-specific log(HR)'s decay to close to 0 by year 8 or 10 (Table 1 in the main manuscript). Additional scenarios S10-S12 in Supplementary Table S3 reproduce assumptions of scenarios 5, 10 and 11, respectively, but with larger *N*=16,000. Finally, additional scenarios S13-S16 assume *no* unmeasured susceptibility (β_s = 0) in order to evaluate the role of sampling error. All scenarios in Supplementary Table S3 assume a 5-year baseline survival of 0.9 and P(S=1) = 0.5.

Results reported in Supplementary Table S3 include the mean of year-specific log(HR) estimates, i.e. the mean of $\hat{\beta}_A(t)$ for year t. In addition, we show the percentages of the 1,000 samples generated for each scenario for which the year-specific (a) log(HR) estimate and (b) the upper bound of the corresponding 95% confidence interval are below 0. Finally, the last column of the Supplementary Table S3 shows the percentage of samples in which the point estimates of log(HR) for treatment for all three last years (*t* = 8-10) were *consistently* negative. Interpretation of the results reported below is presented in section 3.2 of the main manuscript.

Supplementary Table S3 Detailed results regarding "crossing hazards" for selected scenarios with binary susceptibility

Sc.[1]	N	True β_S (HR)	True β_A (HR)	Mean cumulative incidence, %	Mean estimate overall β_A	Performance measures of $\hat{\beta}_A(t)$	Year 1	Year 2	Year 3	Year 4	Year 5	Year 6	Year 7	Year 8	Year 9	Year 10	Years 8-10 [3]
5	4,000	2.303 (10)	0.693 (2.0)	57.4	0.43	Mean $\hat{\beta}_A(t)$	0.65	0.55	0.45	0.37	0.30	0.24	0.20	0.18	0.16	0.16	-
						% $\hat{\beta}_A(t)$ < 0 [1]	0	0	0	0.2	2.5	7.3	11.4	19.5	19.7	23.0	0.8
						% CI($\hat{\beta}_A(t)$) < 0 [2]	0	0	0	0	0	0	0.1	0	0.1	0	-
10	4,000	2.303 (10)	0.262 (1.3)	53.3	0.17	Mean $\hat{\beta}_A(t)$	0.25	0.23	0.19	0.16	0.13	0.12	0.09	0.09	0.08	0.07	-
						% $\hat{\beta}_A(t)$ < 0 [1]	0.3	1.3	5.5	9.8	18.0	22.1	31.3	32.2	34.0	38.4	4.0
						% CI($\hat{\beta}_A(t)$) < 0 [2]	0	0	0	0	0.2	0.3	0.9	0.5	1.6	1.5	-
11	4,000	2.303 (10)	0.405 (1.5)	54.7	0.26	Mean $\hat{\beta}_A(t)$	0.38	0.34	0.29	0.24	0.20	0.16	0.13	0.11	0.10	0.08	-
						% $\hat{\beta}_A(t)$ < 0 [1]	0	0.1	0.8	2.9	8.6	15.3	21.9	27.0	30.6	36.8	1.9
						% CI($\hat{\beta}_A(t)$) < 0 [2]	0	0	0	0	0	0.3	0.4	0.7	0.1	0.3	-
S10	16,000	2.303 (10)	0.693 (2.0)	57.4	0.43	Mean $\hat{\beta}_A(t)$	0.65	0.55	0.45	0.37	0.29	0.24	0.20	0.18	0.17	0.16	-
						% $\hat{\beta}_A(t)$ < 0 [1]	0	0	0	0	0	0.1	1.0	3.8	5.4	6.0	0.1
						% CI($\hat{\beta}_A(t)$) < 0 [2]	0	0	0	0	0	0	0	0	0	0.1	-
S11	16,000	2.303 (10)	0.262 (1.3)	53.3	0.17	Mean $\hat{\beta}_A(t)$	0.25	0.22	0.19	0.16	0.14	0.11	0.09	0.07	0.07	0.06	-
						% $\hat{\beta}_A(t)$ < 0 [1]	0	0	0.1	0.3	2.6	7.6	14.8	21.7	25.1	29.9	2.5
						% CI($\hat{\beta}_A(t)$) < 0 [2]	0	0	0	0	0	0	0.1	0.5	0.2	0.7	-
S12	16,000	2.303 (10)	0.405 (1.5)	54.7	0.26	Mean $\hat{\beta}_A(t)$	0.38	0.34	0.28	0.24	0.20	0.16	0.13	0.11	0.10	0.09	-
						% $\hat{\beta}_A(t)$ < 0 [1]	0	0	0	0	0	1.9	7.0	10.0	15.6	19.8	0.3
						% CI($\hat{\beta}_A(t)$) < 0 [2]	0	0	0	0	0	0	0.1	0	0.1	0.2	-
S13	4,000	0 (1.0)	0.262 (1.3)	20.6	0.27	Mean $\hat{\beta}_A(t)$	0.27	0.25	0.27	0.27	0.27	0.26	0.27	0.27	0.28	0.27	-
						% $\hat{\beta}_A(t)$ < 0 [1]	9.7	12.0	10.6	10.3	11.5	12.0	12.5	13.7	12.2	13.9	0.3
						% CI($\hat{\beta}_A(t)$) < 0 [2]	0.1	0.1	0.1	0	0	0	0.1	0	0.2	0.1	-
S14	4,000	0 (1.0)	0.405 (1.5)	22.0	0.41	Mean $\hat{\beta}_A(t)$	0.42	0.40	0.40	0.42	0.40	0.41	0.41	0.40	0.42	0.42	-
						% $\hat{\beta}_A(t)$ < 0 [1]	2.2	2.1	2.4	2.2	3.5	3.3	3.5	3.1	2.8	4.4	0.0
						% CI($\hat{\beta}_A(t)$) < 0 [2]	0	0	0	0	0	0	0	0	0	0	-
S15	16,000	0 (1.0)	0.262 (1.3)	20.6	0.26	Mean $\hat{\beta}_A(t)$	0.26	0.26	0.27	0.26	0.27	0.27	0.26	0.26	0.26	0.26	-
						% $\hat{\beta}_A(t)$ < 0 [1]	0.2	0.6	0.8	1.7	0.8	1.2	0.6	1.6	1.2	1.4	0
						% CI($\hat{\beta}_A(t)$) < 0 [2]	0	0	0	0	0	0	0	0	0.1	0	-
S16	16,000	0 (1.0)	0.405 (1.5)	22.0	0.41	Mean $\hat{\beta}_A(t)$	0.41	0.40	0.40	0.41	0.40	0.40	0.40	0.41	0.41	0.41	-
						% $\hat{\beta}_A(t)$ < 0 [1]	0	0	0	0	0	0	0	0	0	0.1	0
						% CI($\hat{\beta}_A(t)$) < 0 [2]	0	0	0	0	0	0	0	0	0	0	-

CI: confidence interval. HR: hazard ratio. Sc.: scenario.

[1] All scenarios in Supplementary Table S3 have a 5-year baseline survival of 0.9 and P(S=1) = 0.5.

[2] Percentages of the 1,000 samples for which the year-specific estimated log(HR), denoted $\hat{\beta}_A(t)$ for year t, is smaller than 0.

[2] Percentages of the 1,000 samples for which the 95% confidence interval of $\hat{\beta}_A(t)$ for year t has an upper bound smaller than 0.

[3] Percentage of the 1,000 samples in which the point estimates for all three last years (t = 8-10) were consistently negative.

S4. Changes over time in the mean values of a continuous *S* and corresponding changes in year-specific treatment HRs

Supplementary Fig. S2 shows how, for selected scenarios of Table 2 in the main manuscript, the bias and changes over time in the year-specific treatment HR are driven by differential rates of decrease in the mean value of *S* among participants who remain at risk in later years, with the higher-risk treated group (*A*=1) showing steeper declines (lower panels of Supplementary Fig. S2). This pattern, and the reasons thereof, are generally similar to those shown in Fig. 1 in the main manuscript for a binary *S*. However, for a continuous *S*, the discrepancy between the mean *S* in the two treatment groups increases *steadily* with longer follow-up (Supplementary Fig. S2), as opposed to e.g. scenarios 21 and 23 for a binary *S* in the Fig. 1. Indeed, contrary to a binary *S*, a complete depletion of susceptible can never occur for a continuous *S*, where the mean values of *S* for each treatment group decrease *continuously*, but at different rates, until the end of follow-up (lower panels of Supplementary Fig. S2). Accordingly, for a continuous normally distributed *S*, the underestimation bias of year-specific HRs increases monotonically with increasing cumulative incidence of events observed during follow-up (upper panels of Supplementary Fig. S2), as does the bias of the Cox model-based estimates of the overall treatment effect (Table 2 in the main manuscript).

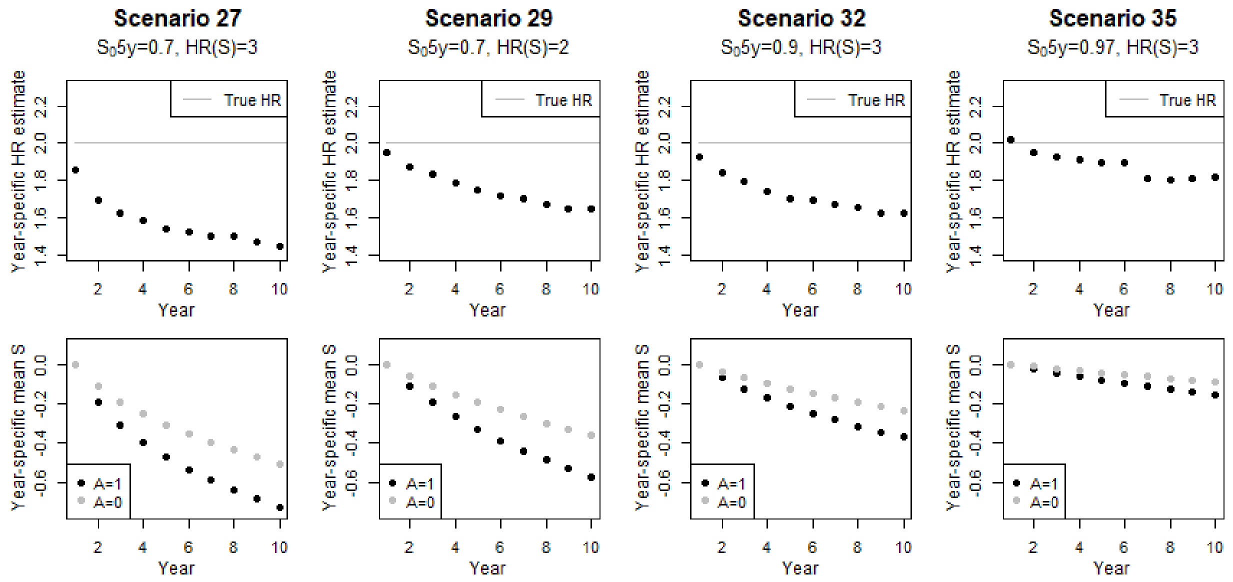

Supplementary Fig. S2 Changes over follow-up time in the (i) bias of year-specific HR estimates for treatment *A* (top panels), and (ii) mean values of *S* among participants who remain at risk until the start of a given year, separately for each treatment group (*A*=1 vs. *A*=0) (bottom panels). Results for four scenarios from Table 2 in the main manuscript, all with a continuous normally distributed *S* and *N*=4,000.

S5. Controlling the number of events in the last period of the trial in targeted simulations of section 4

An important empirical result of the original target trial was that the year-specific HR estimate for treatment effect in the last period of follow-up was as low as 0.70 [1], which suggested a possible protective effect of treatment in this period. This estimate stood in a sharp contrast with the 1st year year-specific HR=1.81 indicating an important risk increase, and was described by Hernán as "crossing hazards"

[2]. Thus, one of the goals of our targeted simulations, in section 4 of the main manuscript, was to explore how strongly an unmeasured susceptibility might have biased the period-specific HR estimate for the last period of this specific trial. More specifically, we explored how low the biased estimate can be, i.e. we were interested in the plausible range of minimum values of the corresponding period-specific estimates, across many simulations. Yet the distribution of the minimum value depends on both the bias and variance of estimates, where the latter – but not the former – is affected by the number of events observed during this period. Thus, in our simulations of section 4 we ensured that the number of events observed in the last period was very similar to the 65 coronary heart disease (CHD) events reported by Manson et al for the same period [1]. This was achieved by first dividing the entire follow-up (until the required 335 events were observed) into 10 equal time intervals, and then combining the last two intervals into what is referred as "the last period of follow-up". As the result, across simulations reported in section 4 of the main manuscript, the corresponding period-specific estimates for the last period were, on average, based on 64-67 events (data not shown), i.e. very close to the 65 events observed in the original trial [1].

S6. Detailed comments about, and reservations against, simulations reported by Stensrud et al [3]

S6.1. Introduction

This section provides focuses on simulations reported by Stensrud et al [3], who attempted to reproduce the salient results of the original hormone therapy randomized-controlled trial (RCT) by Manson et al [1] discussed by Hernán [2]. Below, we summarize the results of Stensrud et al's simulations [3]. We then reconstruct the design of, and the assumptions underlying their simulations. Finally, we present our reservations, and the supporting evidence about their methods and interpretation of their results.

Stensrud et al's simulations combined some characteristics of the original RCT with some additional, rather specific assumptions [3], discussed more in detail below. In their simulations, a failure to adjust for a very strong, rare risk factor (which they refer to as a frailty) resulted in the estimated year-specific HRs for a truly harmful exposure showing a similar pattern to that reported by Manson et al [1]. In particular, in spite of the true treatment HR=1.81 being constant during follow-up, the year-specific estimates decreased below 1.0 after 8 years (Figure 3B in [3]). Thus, in a purely formal sense, the results of these ingenious simulations do demonstrate that it is technically possible to design a set of assumptions under which the dramatic attenuation of the treatment effect in later phases of a trial, such as reported by Manson et al [1] and discussed by Hernán [2], reflects simply a bias due to omitting a very strong risk factor [3].

However, the assumptions underlying simulations reported by Stensrud et al are quite strong and, for the reasons discussed below, we believe that their clinical plausibility is questionable. Below, we reconstruct the assumptions underlying Stensrud et al's simulations [3], and attempt to assess their practical implications as well as to explain our reservations. In addition, in sub-section S6.2, we outline the methods and results of additional simulations we carried out to explore the robustness of Stensrud et al's findings and conclusions.

Each of the following three sub-sections focuses on a specific assumption underlying the simulations reported by Stensrud et al [3].

S6.2. Assuming that 97% of the participants have 0 hazard

Arguably, the most striking assumption imposed by Stensrud et al is that only 3% of the trial participants have a non-zero probability of experiencing a CHD event during the 8 years of follow-up, implying the remaining 97% have a *deterministic* 0 hazard [3]. We are concerned about the clinical plausibility of this assumption. Stensrud et al do *not* provide supporting evidence for this, very strong, assumption [3] and we are not aware of any studies that either supports or makes this assumption. Indeed, e.g. Framingham risk scores suggest that *all* post-menopausal women, even with the absence of any established risk factors, may experience cardiovascular events with a non-zero probability in the next 10 years [4]. Even more pertinent is the fact that Manson et al report explicitly that 4.4% of their trial participants did have a history of previous CHD, stroke, or transient cerebral ischemia [1]. This makes it logically impossible for 97% of the participating women to be deterministically protected against CHD events.

The above reservations become even more important once one realizes that the assumption discussed above has important implications for the results of their simulations. To demonstrate this, we ran two different simulations, both very similar to those reported by Stensrud et al [3] for a heterogeneous treatment effect (see section S6.4 below for details), but in each modifying one particular aspect of their original design. In the first experiment, we relaxed the assumption of strictly 0 hazard for 97% of subjects and, instead, assumed that the 97% "low-susceptibility" subjects have, in fact, a very low but *non-zero hazard*, with untreated women (*A*=0) having cumulative incidence over 7 years of only 1%. (Accordingly, those treated have the corresponding, very low, hazard multiplied by their individual HR, with the mean of 1.81.)

Then, our second experiment was motivated by the observation that, by assuming a hazard of 0 for 97% of the participants, Stensrud et al implicitly redefine the true treatment effect. Whereas they state that true mean HR(A)=1.81 [3], in fact this does *not* apply to 97% of the treated "non-susceptible" women for whom their individual HR (with the mean of 1.81) is multiplied by 0, implying the same zero hazard as for 97% of the non-susceptible untreated participants. To underscore the impact of this (unstated) implicit assumption, we ran the second simulation, in which data were generated exactly as in the original simulations by Stensrud et al, but the analyses were restricted to the 3% of "susceptible" women (treated or untreated), i.e. only to those with non-zero hazards.

Supplementary Table S4 contrasts the year-specific HRs obtained, for a very large sample (N=10^7 as in Stensrud et al's [3] simulations) using (i) their original assumption with 0 hazard for 97% of the participants (2nd column) *versus* (ii) our first modified design with 0.01 cumulative 7-year incidence for 97% of untreated women (3rd column), and (iii) our second simulation, with analyses were restricted to the 3% of susceptible women (4th column). Interestingly, the modified design (ii) shows considerably smaller changes in treatment HRs that decrease from the initial 1.81 to only about 1.34-1.37 in the last four years of the simulated trial, with no evidence of even approaching crossing hazards. (iii) Furthermore, HRs estimated using only data from the 3% susceptible women do show a gradual decrease with longer follow-up (last column of Supplementary Table S4), but they do *not* suggest crossing hazards either, reaching the minimum of 1.24 by year 7, in a sharp contrast to 0.90 for the original Stensrud et al's [3] design (2nd column of Supplementary Table S4). Thus, both our experiments demonstrate clearly that the questionable assumption of 0 hazard for the vast majority of the women is *essential* for the results of Stensrud et al's simulations [3] to approximate the pattern of time-varying changes in treatment HR observed in the hormone therapy trial by Manson et al [1].

Supplementary Table S4 Impact of relaxing Stensrud et al's assumption of 0 hazard for 97% of trial participants: Contrasts between year-specific hazard ratios estimated for each of the three simulated scenarios

Year	Original Stensrud's simulations heterogeneous treatment effect (97% have 0 hazard)	7-year probability of event for 97% "low-susceptibility" women increased from 0 to 0.01	Analysis limited to 3% subset with non-0 hazard
1	1.78	1.78	1.78
2	1.38	1.52	1.53
3	1.21	1.41	1.42
4	1.10	1.36	1.37
5	1.03	1.37	1.33
6	0.97	1.34	1.29
7	0.90	1.35	1.24

S6.3. Assuming high impact of susceptibility

Stensrud et al do *not* explain why they chose to generate a frailty from a compound Poisson model with specific parameter values, and do not present the resulting range of individual hazards [3]. To assess the latter, we generated 10,000,000 values from the distribution they assumed. The resulting non-zero hazards varied from 1.17 to 182.7, with the mean of 33.4, median of 30.8, and standard deviation of 16.0. Thus, the proposed distributional assumption implies a high variance of individual hazards, even among the 3% of susceptible women. The plausibility of such big individual differences is difficult to assess. Together with the reservations explained in sections S6.2 and S6.4, this feature makes it even more difficult to establish the practical relevance of the results of simulations reported by Stensrud et al [3].

S6.4. Assuming treatment effect heterogeneity

In the "heterogeneous treatment" scenario presented by Stensrud et al, which yields results more similar to those reported by Manson et al [1], individual treatment HR is generated from the distribution Gamma(μ=0.81, δ=1.61)+1 [3]. This is a very skewed distribution, and while the mean value of 1.81 agrees with the 1st year estimate from the original trial [1], (a) the median of 1.4 is much lower, and (b) individual values of treatment HRs, among those treated (*A*=1), vary from 1 to about 24.9. Yet, Stensrud et al do *not* provide any rationale for assuming such important heterogeneity [3]. Equally important, treatment heterogeneity logically implies that the estimated treatment HR will vary over follow-up. With harmful true treatment effect (HR(A) > 1.0), individuals with higher treatment HR will tend to have events earlier, while later events among the treated will mostly involve participants with relatively weaker treatment effect, i.e. lower hazards. Thus, time-varying changes in treatment HR estimates reported by Stensrud et al [3] reflect in fact a compound effect of (i) unmeasured susceptibility and (ii) treatment heterogeneity, both acting toward weakening of the harmful effect with longer follow-up.

S6.5. Conclusions

In summary, whereas the results of ingenious simulations by Stensrud et al do demonstrate that it is technically possible to construct a set of assumptions under which the strong attenuation of the estimated treatment reflects simply a bias due to omitting a very important risk factor [3], the underlying

assumptions are quite complex and their clinical plausibility is questionable, at least for cardiovascular events on which the RCT by Manson et al [1] focuses. Equally important, as demonstrated by our experiments summarized in Supplementary Table S4, their simulation results depend critically on these strong assumptions.

S7. Time-dependent association of age at diagnosis with head and neck cancer mortality

In section 5.3 of the manuscript, we present results for the association of cancer stage with cancer related mortality among people diagnosed with head and neck cancer between 2004 and 2017 in the SEER (Surveillance, Epidemiology, and End Results) registries. Below, we present the results of similar analyses of SEER registry data that focus on the association of age at diagnosis with mortality due to head and neck cancer. Supplementary Fig. S3 shows adjusted time-dependent HR estimates for different age groups, relative to the reference of 60-64 years. The hazards increase monotonically from the youngest to the oldest group. However, the HRs for different age groups vary over time, indicating violation of the PH assumption (Supplementary Fig. S3). While we do not have an explicit explanation of these time-dependent estimates we have some clinically plausible conjectures. Older age has the strongest impact on very early mortality (Supplementary Fig. S3a), which may be related to post-surgical complications after the cancer resection, to which older patients are more vulnerable [5, 6]. Furthermore, older patients may be less able to tolerate the most aggressive/effective treatments, and thus may not receive them, or may have problems completing them. Then, the effect of age decreases considerably for 1-3 years after diagnosis, when cancer mortality is mostly driven by cancer-related factors such as cancer stage. Finally, later increases in the impact of older age, 4-10 years after diagnosis, may partly reflect misdiagnosis of some deaths truly due to other natural causes, as due to cancer. Given our simulation results in section 3, these time-varying patterns of changes in HRs for age, observed also for other cancers [7], are very unlikely to be mostly driven by an unmeasured susceptibility. Indeed, Supplementary Fig. S3b shows that time-dependent HRs for age remain almost unchanged after omitting the cancer stage (relative to estimates in Supplementary Fig. S3a, which are adjusted for stage), even if cancer stage a very strong predictor of deaths due to cancer [5, 7, 8]. This finding is in line with our simulation results indicating that the bias due to unmeasured frailty is often quite small.

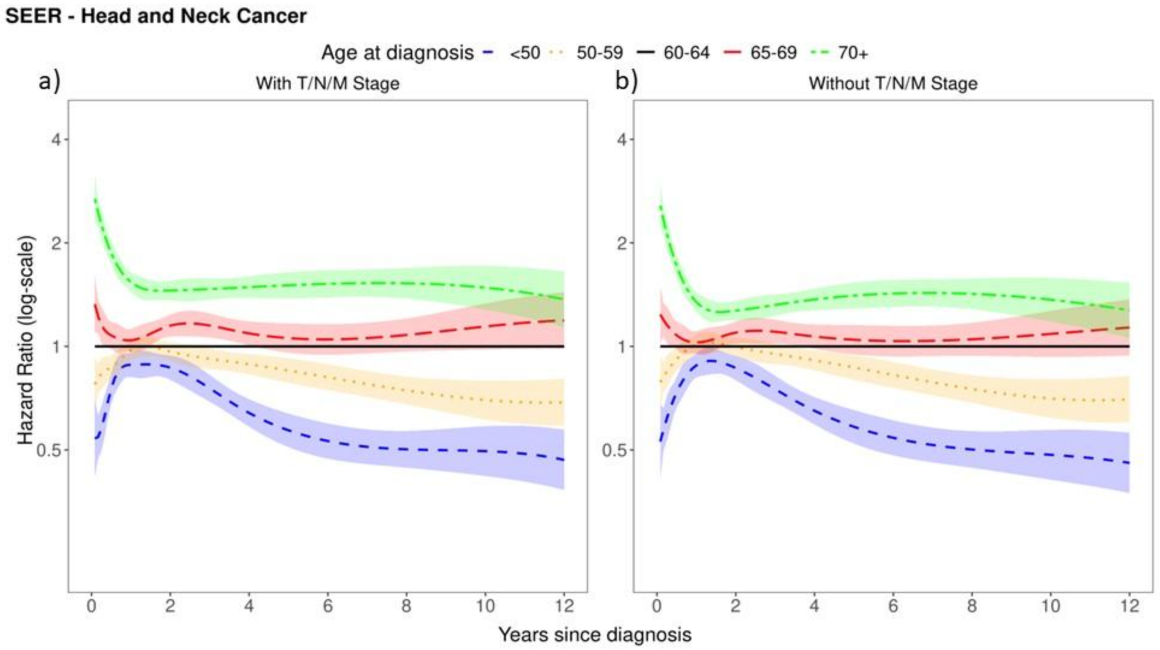

Fig. S3. Time-dependent hazard ratio estimates for different age groups, relative to 60-64 years, for their associations with the hazard of cancer-related mortality, among persons diagnosed with head and neck cancer between 2004 and 2017 in the SEER (Surveillance, Epidemiology, and End Results) registries. Panel a) presents estimates adjusted for cancer T, N and M stage, while in panel b) estimates are *not* adjusted for stage.

References

1. Manson JE, Hsia J, Johnson KC, et al. Estrogen plus progestin and the risk of coronary heart disease. N Engl J Med. 2003;349(6):523-34. https://doi.org/10.1056/NEJMoa030808

2. Hernán MA. The hazards of hazard ratios. Epidemiology. 2010;21(1):13-5. https://doi.org/10.1097/EDE.0b013e3181c1ea43

3. Stensrud MJ, Valberg M, Roysland K, Aalen OO. Exploring selection bias by causal frailty models: the magnitude matters. Epidemiology. 2017;28(3):379-86. https://doi.org/10.1097/EDE.0000000000000621

4. D'Agostino RB, Sr., Vasan RS, Pencina MJ, et al. General cardiovascular risk profile for use in primary care: the Framingham Heart Study. Circulation. 2008;117(6):743-53. https://doi.org/10.1161/CIRCULATIONAHA.107.699579

5. Quantin C, Abrahamowicz M, Moreau T, et al. Variation over time of the effects of prognostic factors in a population based study of colon cancer: Comparison of statistical models. Am J Epidemiol. 1999;150(11):1188-200.

6. Mahboubi A, Abrahamowicz M, Giorgi R, Binquet C, Bonithon-Kopp C, Quantin C. Flexible modeling of the effects of continuous prognostic factors in relative survival. Stat Med. 2011;30(12):1351-65. https://doi.org/10.1002/sim.4208

7. Roberts EK, Luo L, Mondul AM, et al. Time-varying associations of patient and tumor characteristics with cancer survival: an analysis of SEER data across 14 cancer sites, 2004-2017. Cancer Causes Control. 2024. https://doi.org/10.1007/s10552-024-01888-y

8. Luo L, He K, Wu W, Taylor JM. Using information criteria to select smoothing parameters when analyzing survival data with time-varying coefficient hazard models. Stat Methods Med Res. 2023;32(9):1664-79. https://doi.org/10.1177/09622802231181471
